# Global health research and education at medical faculties in Germany

**DOI:** 10.1371/journal.pone.0231302

**Published:** 2020-04-20

**Authors:** Léonie Karduck, Anna Lisa Behnke, Alicia Baier, Dzintars Gotham, Peter Grabitz, Nora Lennartz, Lara Speer, Peter Tinnemann, Walter Bruchhausen

**Affiliations:** 1 Institute for the History, Theory and Ethics of Medicine, RWTH Aachen University, Aachen, Germany; 2 The Berlin Institute of Health, Charité –Universitätsmedizin Berlin, Berlin, Germany; 3 The Berlin Institute of Health, Corporate Member of Freie Universität Berlin, Berlin, Germany; 4 The Berlin Institute of Health, Humboldt-Universität zu Berlin, Berlin, Germany; 5 Universities Allied for Essential Medicines Europe e.V., Berlin, Germany; 6 Independent, Boston, Massachusetts, United States of America; 7 Faculty of Medicine, University of Freiburg, Freiburg, Germany; 8 Institute for Social Medicine, Epidemiology and Health Economics, Germany; 9 Institute for the History and Ethics of Medicine, University of Cologne, Cologne, Germany; 10 Centre for Development Research, University of Bonn, Bonn, Germany; University of Toronto, CANADA

## Abstract

**Background:**

Universities undertake the majority of publicly funded research in Germany and hence bear a responsibility to contribute to global health efforts. So far, involvement and impact of German medical faculties in global health are unknown. Our aim was to systematically asses and evaluate German medical faculties’ contribution to global health related research and education, as well as their policies and practices concerning open access publishing and equitable licensing.

**Methods:**

We assessed the involvement in global health of all 36 publicly funded medical faculties in Germany during 2010–2014 in three areas: innovation, access and education, using the following indicators: research funding and publications focused on global health or poverty-related and neglected diseases; open access publishing and policies promoting access to medical innovations worldwide; provision of global health education. Data were gathered from public databases, university websites and questionnaires sent to individual universities for validation and triangulation.

**Results:**

There was a high level of variability between institutions and indicators. The proportion of research funding for poverty-related and neglected diseases research ranged between 0.0–1.1%. The top five institutions received nearly 85% of the total poverty-related and neglected diseases research funding. 20 of 36 universities had an institutional open access publishing policy, 19 had an open access publishing fund, 16 had neither. Only one university reported having used an equitable licensing policy. 22 of 36 faculties provided some global health education, but only one of them included global health in their core undergraduate medical curriculum as a compulsory course with more than just single lectures.

**Conclusion:**

Obtained data indicate that global health and poverty-related and neglected diseases research at German medical faculties is highly concentrated in a few institutions, open-access publishing and equitable licensing policies are mostly absent, and only little global health education exists. Universities and government should address global health strategically in both research and education at medical faculties to reflect the country’s economic and political weight and human resource potential.

## Introduction

An estimated half of the world’s population lacks access to medicines [1]. Many of the most pressing health needs in low- and middle-income countries have been historically neglected in health research. Compounding these and other challenges in global health, there is little knowledge about global health among many scientists and clinicians. Publicly funded universities are in a position to address these issues, by including global health teaching in their curricula, increasing research efforts in historically neglected areas, and ensuring that medicines and other health products developed through their research are available affordably in the countries with the greatest health needs.

Since universities undertake the majority of publicly funded research in Germany, the funding public expects them to share their contributions to global health responsibly [2]. No systematic assessment of global health related research at German universities however has been conducted so far. Recent studies assessing global health research at universities in the United States of America (USA), Canada, and the United Kingdom (UK) identified significant shortcomings in the equitable dissemination of research results [3–5]. In terms of the share of health research funding compared to corresponding global burden of disease, research on poverty-related and neglected diseases is underfinanced by a factor of five at universities in the UK [5]. Similar imbalance in the allocation of resources for neglected diseases research were found for North American research universities [6].

Key tools for removing barriers for access to certain health services through licensing and patenting in low- and middle-income countries and making research products, inventions and knowledge accessible for all people, are open access publishing, but also policies for the equitable licensing of intellectual property [7–10]. Equitable licensing models (also referred to as socially responsible licensing or humanitarian licensing) can facilitate access to health technologies (e.g. medicines, vaccines and diagnostics) developed at universities for people in low- and middle-income countries [11, 12].

Our study aim is to systematically assess and evaluate German medical faculties’ measurable contributions to global health by systematically assessing global health research, measuring global health related grants and publications, university policies and practices relating to open access publishing and equitable licensing, and global health education.

This research project was conducted by the student organizations Universities Allied for Essential Medicines (UAEM) Germany in cooperation with the German Medical Students’ Association (bvmd). Selected data from this analysis has been published online on an interactive website, available at www.globale-gesundheit.de.

## Methods

The study is divided in three parts: (1) global health innovation, sub-divided in (1.1) research funding and (1.2) publications, (2) global health access, including open access publishing and equitable licensing and (3) global health education.

### Study design

We collected, reviewed and analysed publicly available secondary data across 36 publicly funded German medical faculties.

### Setting

German medical faculties are in general, exclusively publicly funded medical schools, except the Medical School Hamburg - University of Applied Sciences and Medical University, the Brandenburg Medical School Theodor Fontane and the medical school of the Witten/Herdecke University.

The total number of all 36 medical schools students enrolled to study human medicine as major subject from 2010 to 2014 was on average about 80.000 students per year. The number of students per medical faculty varied from a minimum of below 200 (Oldenburg) to a maximum of around 6100 (Berlin) students (see detailed data in Appendix).

Medical education at public medical schools is exclusively funded by 15 of the 16 German federal states (Bundesländer), whereas the city state of Bremen does not have a medical school. Their research, besides some limited university funding for personnel, equipment and supply, is mostly funded by extramural third-party funds, e.g. the German Research Foundation (Deutsche Forschungsgemeinschaft, DFG), programs of the German Federal Ministry for Education and Research (Bundesministerium für Bildung und Forschung, BMBF), private foundations such as Volkswagen Stiftung or European Commission programmes. Thus, medical schools influence their own research agenda e.g. by selecting relevant staff among applicants for faculty positions, by creating new departments, or other faculty entities, or by providing incentives - but overall medical faculties do not directly fund research activities themselves.

Being part of public universities, medical faculties are expected to implement open access and intellectual property licensing policies of their respective university or federal state.

The time period analyzed was five years, from 1 January 2010 to 31 December 2014. The end of 2014 was considered the most recent feasible cut-off in view of data availability.

### Data management

Collected data were coded independently by two reviewers, each blinded to the selection of the other. Coding for each search result was compared and discrepancies were resolved by consensus. We provide an overview of data gathered by university (see S1 Table). All collected variables that could be answered with ‘yes’ or ‘no’ were coded as ‘present’ or ‘absent’.

### Ethics statement

All data gathered and analysed within this study are publicly available secondary data, therefore an ethics committee statement is not required.

All individuals responding to invitations by email, letter or website to contribute secondary data were informed about the study intent and that personalized data is anonymized. By providing data they confirmed having read the information on the study intent and agreed to the outlined use of the data they provide.

## Part: 1. Global health innovation

Poverty-related and neglected diseases were defined based on G-FINDER 2014 criteria [13]. Low- and middle-income countries were defined by World Bank classification [14]. The Consortium of Universities for Global Health definition for ‘global health’ was used [15].

### Search strategy

The search was based on names of low- and middle-income countries and global health referred items; the full search term is available in the supporting information (see S2 Appendix).

### Statistical methods

Statistical tests were calculated using SPSS Statistics (version 24) and Microsoft Excel (version 15.0.5067.1000).

### Data excluded

The Carl von Ossietzky University of Oldenburg was not assessed in the Innovation part of this study, as the medical faculty was only established in 2012 and thus comparable analysis was not possible.

### 1.1. Research funding

#### Setting

The percentage of the overall medical research funding attributable to research with a focus on global health and poverty-related and neglected diseases was calculated. Third-party research funds which covers the vast majority of means for research at German medical faculties are external research funds that are provided, for example, in the form of project-specific grants, by public or private entities separately from core funding for the regular university budget [16]. The amount of third-party research funds is often used as an indicator of research activity [17]. Research projects starting between January 1, 2010 and December 31, 2014 were included.

#### Data source/collection

Data on third-party research funds were collected from the public databases of five major (global health) research funders in Germany: the German Federal Ministry of Education and Research, the German Research Foundation, the European Commission, the Bill and Melinda Gates Foundation and the Volkswagen Foundation.

Questionnaires were sent by email to vice deans of research to seek data on third-party research funding that may not have been captured through analysis of grant databases and to validate information received from database searches (triangulation). Additional third-party research funding was identified through direct communication in only two cases–for the medical faculties of the University of Greifswald and the University of Bonn (see S1 Appendix).

#### Search strategy

The search strategy was tailored to accommodate for differences in database search tools. To identify grants made to each faculty, database searches were first filtered by ‘executive authority’ (the German Federal Ministry of Education and Research), ‘institution’ (the Volkswagen Foundation), or ‘location of the medical faculty’ (the Bill and Melinda Gates Foundation). Projects from the European Commission CORDIS database were extracted by a CORDIS service desk worker. In the German Research Foundation database, a search term was used that included terms covering poverty-related and neglected diseases, low- and middle-income countries, and global health topics, as well as the city names of all medical faculties.

#### Data management

Grants identified as described above were then manually coded according to medical faculty affiliation, time frame, and global health or poverty-related and neglected diseases relevance. The categories ‘global health’ and ‘poverty-related and neglected diseases’ were mutually exclusive. Research on biopsychosocial health, considering social, political, economic, cultural and environmental determinants and focusing on global health equity was defined as global health relevant, whereas research investigating the 34 neglected diseases of the G-Finder 2014 as poverty-related and neglected diseases relevant.

#### Statistical analysis

With global health/poverty-related and neglected diseases-attributable grants making up the numerator, the denominator was the total research funding of funds each institution received during the study period as provided by the ‘landkarte-hochschulmedizin’ database maintained by the German Medical Faculty Association [18].

Spearman’s correlation coefficient was used to test the hypothesis that increased total institutional third-party research funds show positive correlation with third-party research funds for global health and third-party research funds for poverty-related and neglected diseases after checking for normal distribution with the Shapiro-Wilk test [19, 20].

### 1.2. Publications

#### Setting

For each medical faculty, we calculated the proportion of the overall PubMed-listed publications that had a focus on global health and poverty-related and neglected diseases (see S2 Appendix). Publications published between January 1, 2010 and December 31, 2014 were included.

#### Data management

PubMed results were coded for medical faculty affiliation and whether their thematic focus was on global health or poverty-related and neglected diseases.

#### Statistical analysis

The number of global health/poverty-related and neglected diseases attributable publications constitute the numerator, the denominator was the total number of research publications of each medical faculty during the five-year time frame as provided by the ‘landkarte-hochschulmedizin’ database maintained by the German Medical Faculty Association [21].

Lastly, data on research groups, centers, institutes, or professorial chairs in the field of global health and/or poverty-related and neglected diseases at each medical faculty were collected through questionnaires sent to vice deans of research.

## Part 2. Global health access (open access publishing and equitable licensing)

### Setting

Policies regarding open access and equitable licensing are in general governed at the institutional rather than the faculty level, thus in this part of the study we consider and refer to universities instead of medical faculties.

### Collected variables

University policies and practices with regard to open access publishing were evaluated using the following indicators: whether an open access policy exists at the university, the availability of a designated staff member to support researchers seeking open access publishing, whether the university website addresses open access publishing, whether it holds open access events (e.g. seminars, presentations etc.), whether it hosts an open access repository, whether it provides open access fund and whether the university is a signatory of the Berlin Declaration [22]. The ‘Berlin Declaration on Open Access to Knowledge in the Sciences and Humanities’, one of the milestones of the open access movement, fosters open access publishing with the aim of making scholarly research results and cultural heritage freely accessible and usable for scientists and the public [23]. Its mission of disseminating knowledge is only partly completed, if the information is not made widely and readily available to society.

### Data source/collection

Data were obtained through a questionnaire to open access representative of each university, or, if not available, to the medical faculty vice dean of research and supplemented by online searches.We identified open access repositories by reviewing the Directory of open access Repositories (OpenDOAR) and members of the DINI Certificate initiative ‘Open Access Repositories and Publishing Services’ [24, 25]. Data concerning the presence of university open access funds were collected from the German Project Information System (GEPRIS) of the German Research Foundation [26], since most of open access funds were provided within the German Research Foundation’s open access publishing program.

#### Search strategy

Data collection for equitable licensing was done by Google searches using the following terms: “university name” AND "equitable licensing", “Humanitarian licensing”, “soziale Lizenz” (social licence), “gerechte Lizenz” (equitable licence), "soziale Verantwortung" AND “Forschung” (social responsibility AND research), "Lizenzierung" (licensing), "Patentstrategie" (patent strategy), "Patentverwertungsstrategie" (patent exploitation strategy), "Technologietransfer" (technology transfer), "product development partnership", “public private partnership”, "PDP", and “Produktentwicklungspartnerschaft” (product development partnership). A similar search was performed using individual universities websites’ native search function. For each search, the first 20 results were screened regarding licensing strategies and product-development partnerships. Additionally, questionnaires were sent to each university’s technology transfer office.

### Statistical analysis

To assess the percentage of global health- or poverty-related and neglected diseases-focused research articles that were published open access, we calculated the proportion of all global health- or poverty-related and neglected diseases-focused articles identified through PubMed that were also present in PubMed Central’s open access subset (https://www.ncbi.nlm.nih.gov/pmc/tools/openftlist/).

## Part 3. Global health education

### Collected variables

Results from all sources were merged for each medical faculty and four components were extracted from the data: 1. Global health teaching at the medical faculty, 2. Cooperation with other faculties within the university for global health teaching, 3. Student exchange programs in cooperation with low- and middle-income countries and 4. Clinical or research cooperation with low- and middle-income countries institutions.

Global health teaching was analyzed and evaluated by different criteria: availability, quantity, type and frequency. Student exchange programs with low- and middle-income countries included both incoming and outgoing programs, and only verifiably active programs were included.

### Data source/ collection

Global health education was analyzed by a standardized search of the university website, as well as by questionnaires sent to the vice dean of education of each medical faculty and to German medical students using the e-mail distribution list of the German medical students’ association. Additionally, unpublished data provided by the Global Health Alliance in Germany, an initiative of lecturers and students at medical faculties, were used to identify contact persons for global health education at medical faculties in Germany, who were asked for further information on global health education at their medical faculties.

An online search on co-operations with low- and middle-income countries was done on the web pages of the German Corporation for International Cooperation (GIZ), the German Academic Exchange Service (DAAD), and the Else-Kröner-Fresenius Foundation. These organizations encourage and promote cooperation programs between Germany and low- and middle-income countries.

### Search strategy

An online search was performed for medical faculties with missing responses to the questionnaires, using the university webpage’s search function including the following search terms in English and German: “Globale Gesundheit” (global health), “Internationale Gesundheit” (international health), “public health”, “Vernachlässigte Krankheiten” (neglected diseases), “neglected tropical diseases”, and “Tropenmedizin” (tropical medicine). The first ten results of each search were screened and analyzed. We noted occasional search results pertaining to faculties other than the medical faculty, but these results were not included in the study.

## Results

Four of 35 medical faculties answered on third-party research funds. Data on open access publishing could be gathered from 16 of 36 universities while only seven universities responded to equitable licensing data. Information on global health education was gathered from 222 students from 25 of the 36 medical faculties (1–44 responses per institution). Thirteen of 36 medical faculties replied to our global health education questionnaire.

## Part 1. Global health innovation

### 1.1. Research funding

The 35 of 36 medical faculties included in this part of the study received a total amount of 7.8 billion EUR of third-party research funds over 2010–2014 [18], of which 11.6 million EUR (0.2%) could be found for global health research and 17.3 million EUR (0.2%) for poverty-related and neglected diseases research (Table 1).

**Table 1:** Absolute count of global health and poverty-related and neglected diseases third-party research funds (in Euro) at surveyed medical faculties in Germany from 2010–2014, ranked by poverty-related and neglected diseases.

Medical faculty of	GH	PRND
University of Tübingen	1,158,778 €	4,345,199 €
Heidelberg University (Medical Faculty Heidelberg)	841,602 €	3,482,441 €
Ludwig Maximilians University of Munich	1,036,097 €	3,144,813 €
University of Bonn	809,215 €	1,885,887 €
Friedrich-Alexander University Erlangen-Nürnberg	- €	1,842,764 €
University of Leipzig	- €	768,000 €
Johannes Gutenberg University of Mainz	487,922 €	391,593 €
Otto-von-Guericke University Magdeburg	399,346 €	301,954 €
Hannover Medical School	- €	295,200 €
Heinrich-Heine University Düsseldorf	536,387 €	246,950 €
Ruhr University Bochum	- €	217,890 €
University of Ulm	- €	115,153 €
Charité - Universitätsmedizin Berlin	1,216,819 €	81,434 €
University of Giessen	- €	74,978 €
Technical university of Munich	599,890 €	52,935 €
University of Würzburg	- €	45,000 €
University of Münster	86,400 €	21,607 €
University of Lübeck	247,829 €	18,810 €
University of Saarland	1,173,715 €	- €
University of Greifswald	770,365 €	- €
University of Freiburg	652,550 €	- €
University of Rostock	409,921 €	- €
Martin Luther University of Halle-Wittenberg	260,395 €	- €
University of Cologne	234,330 €	- €
University of Aachen	172,000 €	- €
University of Duisburg-Essen	170,000 €	- €
University of Hamburg	134,650 €	- €
Dresden University of Technology	84,104 €	- €
University of Göttingen	40,898 €	- €
Friedrich Schiller University Jena	23,540 €	- €
Christian Albrechts University Kiel	22,194 €	- €
University of Regensburg	9,943 €	- €
Goethe University Frankfurt	- €	- €
Heidelberg University (Medical Faculty Mannheim)	- €	- €
Philipps University of Marburg	- €	- €
Total	11,578,890 €	17,332,608 €

GH: global health.

PRND: poverty-related and neglected diseases.

**Fig 1:**
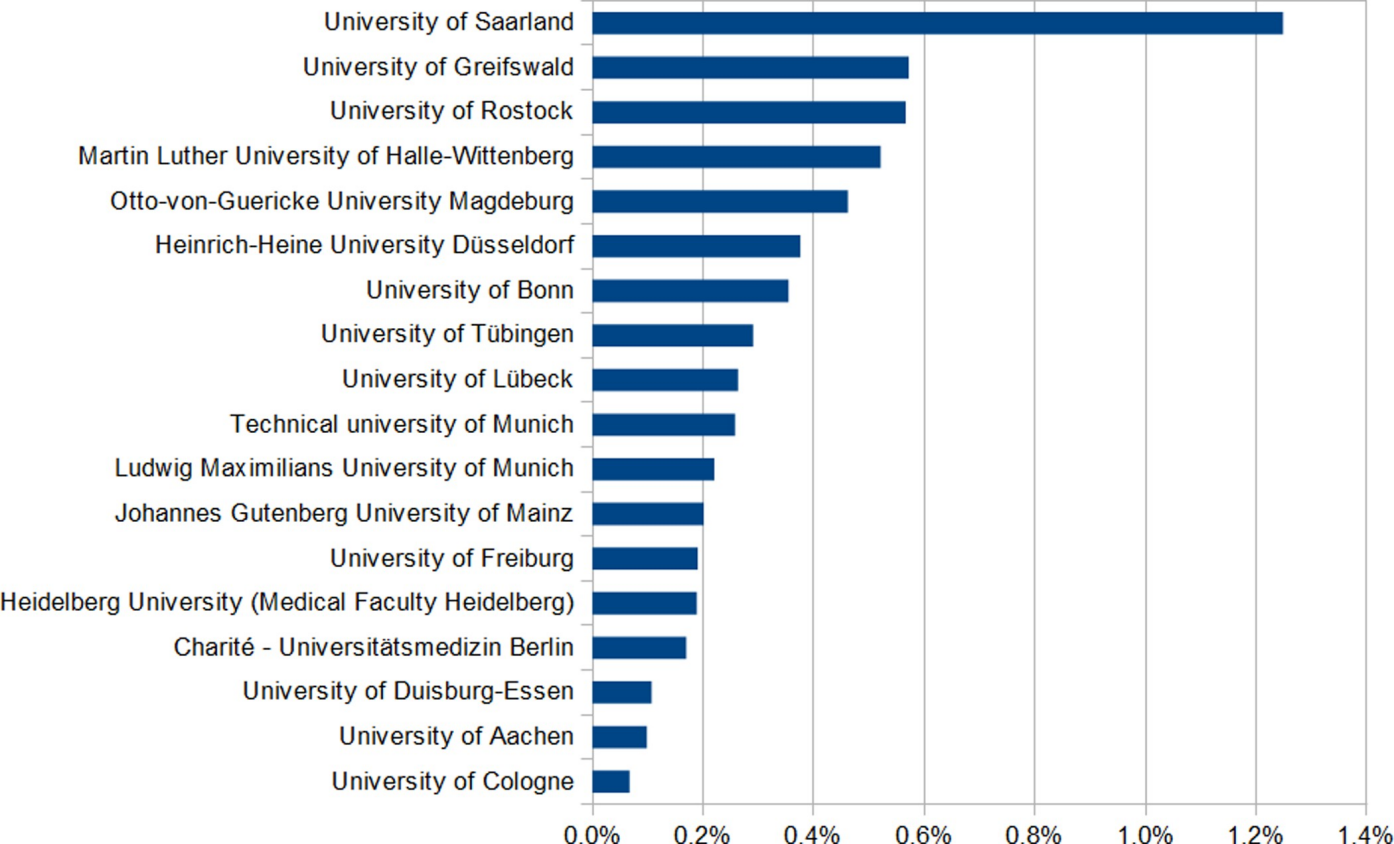
Proportion of global health third-party research funds at medical faculties in Germany, 2010–2014. Medical faculties of Goethe University Frankfurt, Heidelberg University (Medical Faculty Mannheim), Philipps University of Marburg, University of Würzburg, University of Giessen, University of Ulm, Hannover Medical School, Ruhr University Bochum, University of Leipzig, Friedrich-Alexander University Erlangen-Nürnberg, University of Regensburg, Christian Albrechts University Kiel, University of Göttingen, Friedrich Schiller University Jena, University of Münster, Dresden University of Technology, University of Hamburg had a global health share below 0.1%.

**Fig 2:**
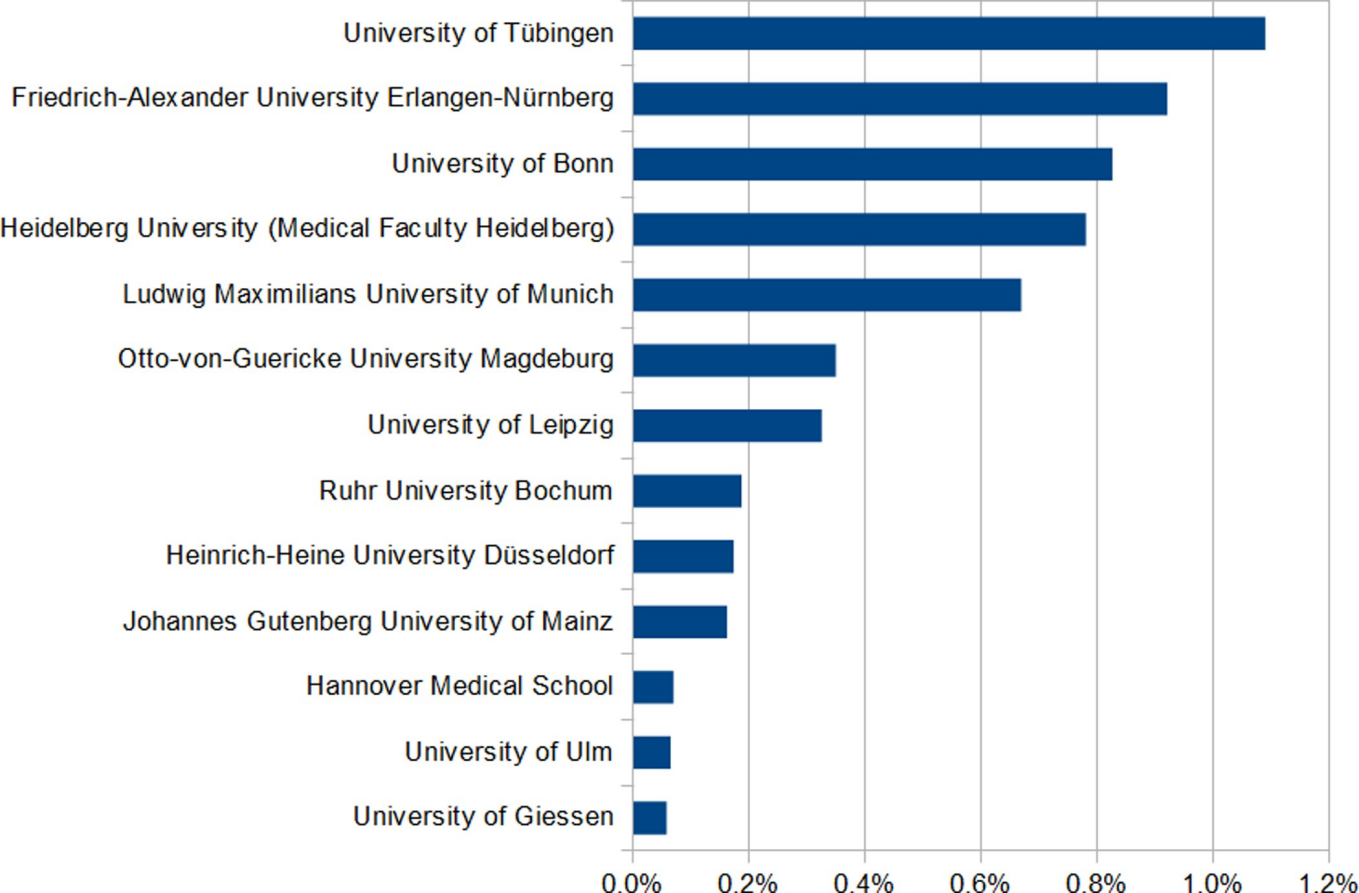
Proportion of poverty-related and neglected diseases third-party research funds at medical faculties in Germany, 2010–2014. Medical faculties of Goethe University Frankfurt, Heidelberg University (Medical Faculty Mannheim), Philipps University of Marburg, University of Regensburg, Christian Albrechts University Kiel, University of Göttingen, Friedrich Schiller University Jena, Dresden University of Technology, University of Hamburg, University of Cologne, University of Aachen, University of Duisburg-Essen, University of Freiburg, Martin Luther University of Halle-Wittenberg, University of Rostock, University of Greifswald, University of Saarland, University of Münster, Charité - Universitätsmedizin Berlin, University of Lübeck, University of Würzburg, Technical university of Munich had a global health share below 0.1%.

The median proportion of third-party research funds attributable to global health research was 0.1% (interquartile range 0.0–0.3%), to poverty-related and neglected diseases research 0.0% (interquartile range 0.0–0.2%). The proportions of third-party research funds attributable to global health and poverty-related and neglected diseases research for each medical faculty are shown in Figs 1 and 2.

Poverty-related and neglected diseases research funding exceeded 1% total third-party research funds in a single case (medical faculty of the University of Tübingen).

Ten of the 35 medical faculties (29%) had no global health-attributable research funding and 17 medical faculties (49%) had no poverty-related and neglected diseases-attributable research funding. The top five institutions in terms of absolute global health funding represented 47% of all global health research funding and 85% of all poverty-related and neglected diseases research funding. There was non-significant correlation between third-party research funds on global health and the total amount of the institutions’ third-party research funds (*r* = 0.22, *p* = 0.199), but significant moderate positive correlation between third-party research funds on poverty-related and neglected diseases and the total amount of third-party research funds of the institution (*r* = 0.40, *p* = 0.017). The major share of global health funding as well as poverty-related and neglected diseases funding was granted by the German Federal Ministry for Education and Research (13,685,168 €), followed second by DFG (6,476,020€) and third BMGF (3,277,300€).

### 1.2 Publications

**Fig 3:**
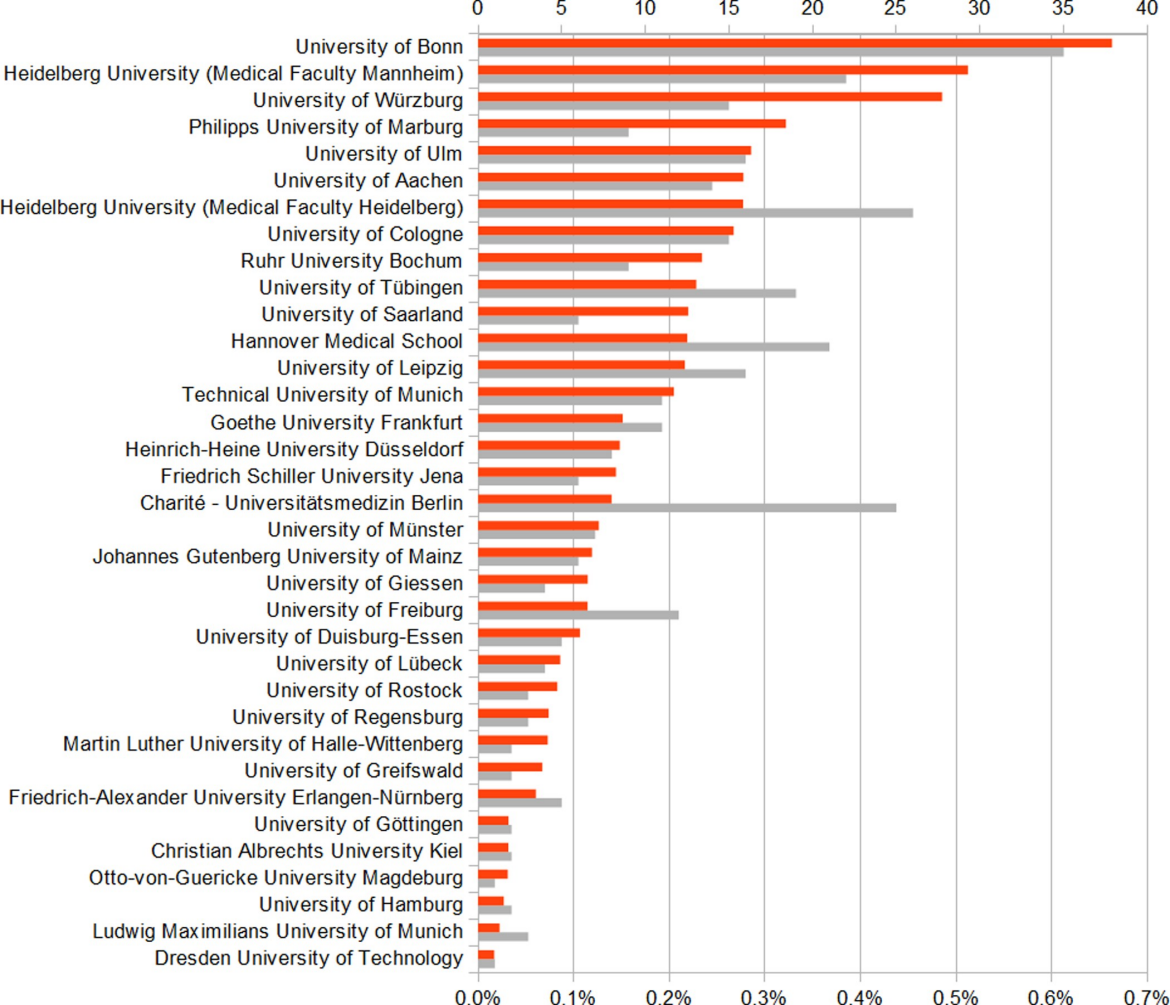
Absolute number and relative proportion of global health publications per medical faculty, 2010–2014. Overall number of global health publications: n = 348, x-axis: Orange: Proportion of global health publications relative to total number of publications (percentage), Grey: Absolute number of global health publications, y-axis: medical faculty.

**Fig 4:**
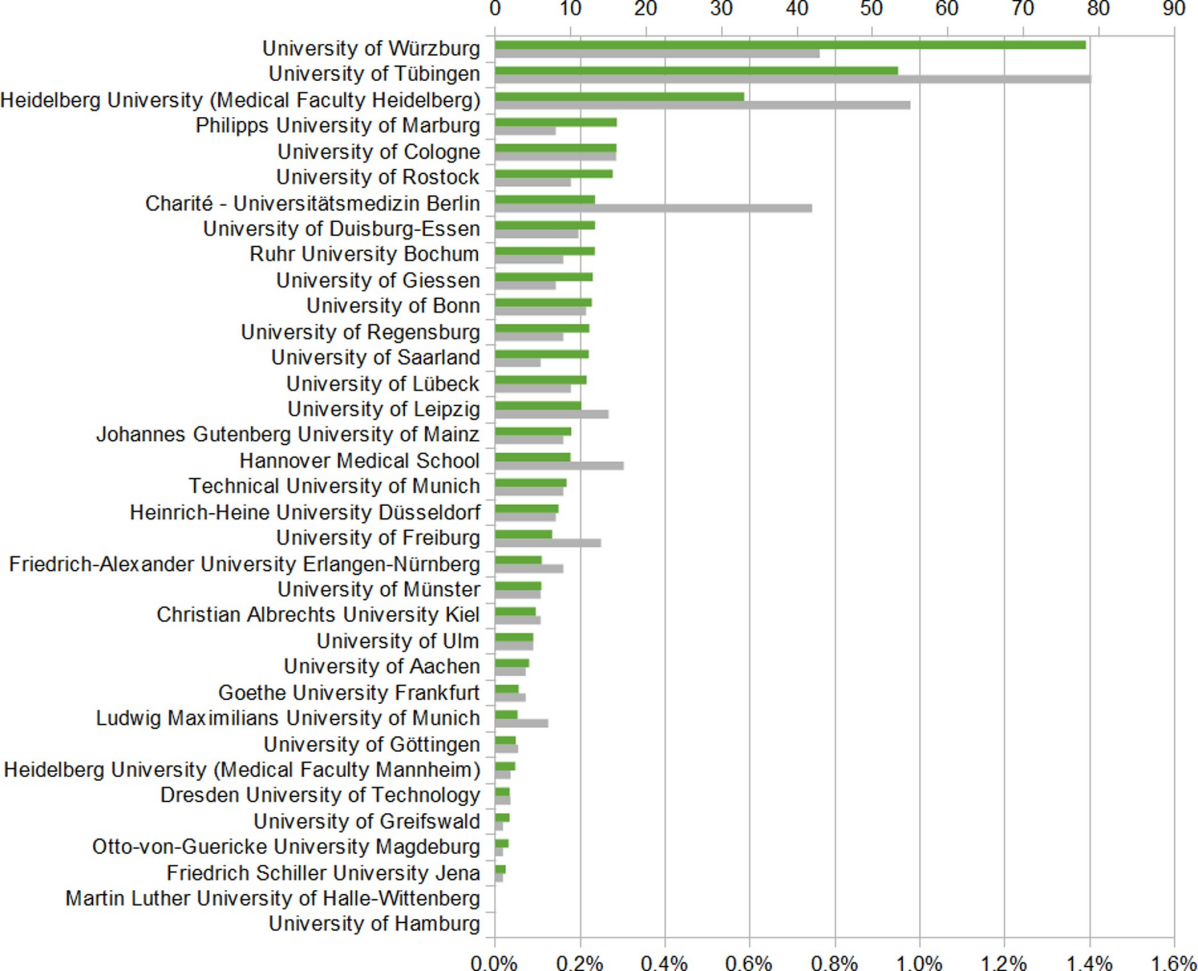
Absolute number and relative proportion of poverty-related and neglected diseases publications per medical faculty, 2010–2014. Overall number of poverty-related and neglected diseases publications: n = 441, Green: Proportion of poverty-related and neglected diseases publications relative to total number of publications (percentage), Grey: Absolute number of poverty-related and neglected diseases publications, y-axis: medical faculty.

Of the 211,236 publications published over 2010–2014 at the 35 medical faculties included in this study [21], 0.2% were related to global health and as well to poverty-related and neglected diseases. A median of 0.1% (interquartile range 0.1–0.2%) of all publications were categorized as global health and 0.2% (interquartile range 0.0–0.2%) as poverty-related and neglected diseases. The proportion of research publications focused on poverty-related and neglected diseases exceed 1% in a single case (medical faculty of the University of Würzburg) (Figs 3 and 4).

## Part 2. Global health access

Ten of 36 universities (28%) had signed the ‘Berlin Declaration on Open Access to Knowledge in the Sciences and Humanities’ and 20 had an institutional open access publishing policy (S2 Table).

Fifteen universities (42%) had neither. Nineteen universities (53%) had an open access publishing fund in 2014 (S2 Table). Nineteen universities (53%) had an open access representative and 25 universities (69%) provided open access information on their webpage. Nineteen universities (53%) offered open access events, such as seminars, 11 of them did so regularly. Twenty-five institutions (69%) having a repository including English articles were identified; 20 of them with an interface in English and five of them with a German interface only. Nine universities (25%) hosted an open access journal. A median proportion of 31.6% (interquartile range 23.1–43.2%) of global health or poverty-related and neglected diseases publications was published open access with a maximum of 60.0% at the University of Göttingen and a minimum of 5.0% at the Technical University of Munich.

Three institutions (8%) had equitable licensing policies. Of these, only the University Medical Center Hamburg-Eppendorf, which has an equitable technology transfer policy at the medical faculty level, reported having employed the policy in licensing a health technology between 2010 and 2014 (Table 2).

**Table 2:** Overview of equitable licensing from 2010–2014.

University	Policy	Equitable Licensing	Patents in LMICs
University Medical Center Hamburg-Eppendorf	policy preferring equitable licensing	at least once usage of equitable licensing	less than 20% of innovations patented in LMICs
University of Münster	policy preferring equitable licensing	no commitment	no response
University of Tübingen	policy preferring equitable licensing	no commitment	no response
Charité - Universitätsmedizin Berlin	no policy^a^	no commitment	less than 20% of innovations patented in LMICs
Friedrich-Alexander University Erlangen-Nürnberg	no policy^a^	no commitment	no response
Philipps University of Marburg	no policy	no commitment	less than 20% of innovations patented in LMICs
University of Aachen, Ruhr University Bochum, University of Bonn, Dresden University of Technology, Heinrich-Heine University Düsseldorf, University of Duisburg-Essen, Goethe University Frankfurt, University of Freiburg, University of Giessen, University of Göttingen, University of Greifswald, Martin Luther University of Halle-Wittenberg, Hannover Medical School, Heidelberg University (Medical Faculty Heidelberg), University of Saarland, Friedrich Schiller University Jena, Christian Albrechts University Kiel, University of Cologne, University of Leipzig, University of Lübeck, Otto-von-Guericke University Magdeburg, Heidelberg University (Medical Faculty Mannheim), Johannes Gutenberg University of Mainz, Ludwig Maximilians University of Munich, Technical University of Munich, Carl von Ossietzky University of Oldenburg, University of Regensburg, University of Rostock, University of Ulm, University of Würzburg	no policy	no commitment	no response

**a** No policy but information and considerations about equitable licensing.

LMICs Low- and middle-income countries.

## Part 3. Global health education

Twenty-two of the 36 of medical faculties (61%) provided some global health education, apart from two, all consisting of elective courses. Fourteen of the 36 (39%) offered no courses, or information was not available (Table 3).

**Table 3:** Overview of global health education at medical faculties in Germany during 2010–2014.

Medical faculty of	Global health education?	Regularity^a^?	More than one type^b^ of global health education?	More than two events?	Included in compulsory medical curriculum?
Carl von Ossietzky University of Oldenburg	yes	yes	no	yes	yes
Heidelberg University (Medical Faculty Heidelberg)	yes	yes	yes	yes	no
Ludwig Maximilians University of Munich	yes	yes	yes	yes	no
University of Aachen	yes	yes	yes	yes	no
University of Hamburg	yes	yes	yes	yes	no
University of Giessen	yes	yes	yes	yes	no
University of Ulm	yes	yes	yes	yes	no
University of Würzburg	yes	yes	yes	no	no
Otto-von-Guericke University Magdeburg	yes	no	no	no	yes
University of Bonn, Friedrich Schiller University Jena, Charité - Universitätsmedizin Berlin, Heinrich-Heine University Düsseldorf, University of Münster, University of Freiburg, University of Greifswald, University of Leipzig, Philipps University of Marburg, Martin Luther University of Halle-Wittenberg, Technical University of Munich, University of Regensburg	yes	yes	no	no	no
Dresden University of Technology	yes	no	no	no	no
University of Tübingen, Goethe University Frankfurt, University of Cologne, University of Lübeck, Johannes Gutenberg University of Mainz, University of Rostock, Friedrich-Alexander University Erlangen-Nürnberg, University of Duisburg-Essen, University of Göttingen, Christian Albrechts University Kiel, Ruhr University Bochum, Hannover Medical School, University of Saarland, Heidelberg University (Medical Faculty Mannheim)	no	no	no	no	no

**a** In three or more consecutive years and/or semester.

**b** E.g. excursions, seminars, lectures etc.

no–No global health education or no data available.

Only two medical faculties (6%) had a compulsory global health education as part of its curriculum. While the Carl von Ossietzky University of Oldenburg, which offers a cross-border medical degree program to a small number of students in a German-Dutch collaboration with the University of Groningen, had a compulsory global health course including fifteen lectures within two academic years, the Otto-von-Guericke University Magdeburg had a single global health seminar as part of its curriculum. At four medical faculties (11%) global health courses were run as interdisciplinary courses with other faculties.

Twenty-three medical schools (64%) offered students opportunities to study in low- and middle-income countries (outgoing-programs) but we could only identify incoming-programs for students from low- and middle-income countries at seven medical faculties (19%). Thirteen of 36 institutions (36%) did not have any exchange-programs run in cooperation with low- and middle-income countries.

Nineteen of the 36 medical faculties (53%) offered research and/or training partnerships with hospitals and/or research institutions in low- and middle-income countries.

## Discussion

Germany is a high-income country, with considerable international political weight and an expressed intent of the federal government to prioritize global health, in particular participating in finding solutions in neglected fields of global health and poverty-related and neglected diseases disproportionately affecting low- and middle-income countries [27, 28].

Over the past years, Germany has significantly strengthened its role in global health, for example by prioritizing global health during the German G7 and G20 presidencies and through strategic funding of multilateral organizations involved in global health. To complete the picture of Germanys involvement in global health, and to hold the federal government accountable to the policies they set, it is therefore of importance to understand the extent of Germany’s publicly funded institutions involved in global health and poverty-related and neglected diseases research and higher education. Funding for global health research and open access publishing is mostly provided by the federal government, as the main public funder of research, global health education is mostly financed through federal states universities funding.

Our results, so far unfortunately, show that research activity in global health and poverty-related and neglected diseases in Germany is low. Research funding for poverty-related and neglected diseases is more than 60 times lower than would be expected based on the proportion of the global burden of disease attributable to this disease group (0.2% of research funding versus 13.8% of global burden of disease) [29]. Funding for global health research is at even lower levels. This gap is seen as an opportunity for German research institutions to increase their global impact [30]. As in contrast to US and UK universities, German universities do not have considerable research funding of their own, but are dependent on extramural funding for most of their research. Their extension of global health research is dependent on respective federal funding programs which did not exist so far, but have been implicitly promised by the government and openly demanded by the scientific community for several years. Our findings are in line with those of a recent study by leading experts in the field of neglected tropical diseases funded by the German Ministry of Research and Education. The authors conclude that German research institutions need to urgently receive considerably more funding in this field [31, 32].

We observed a high level of variability across institutions, with nearly half of medical faculties receiving no research funding for poverty-related and neglected diseases, while the top five medical faculties represented 85% of all poverty-related and neglected diseases research funding. The major part of poverty-related and neglected diseases research at medical faculties is concentrated at very few institutions. The question of whether this distribution is desirable should be carefully examined due to a sufficient coverage of research in this field. It is debatable if there should be poverty-related and neglected diseases research at every medical faculty in Germany given the expertise and infrastructure needed; but if a concentration on just a few faculties is intended, this should be strategically decided with the aim of addressing key research gaps in this field.

Global health research publications are more evenly distributed across German medical faculties than poverty-related and neglected diseases research publications. This may reflect the wider range of disciplines generally included in global health, allowing smaller faculties to engage in this field. For research funding, however, our findings showed a higher amount of research funding for poverty-related and neglected diseases than for global health. This may reflect the restriction of our analysis to medical faculties; as global health is a highly interdisciplinary field further studies concerning global health activity at other faculties are needed.

It is encouraging that open access funds, which support the publishing fees generally leveed by open-access (but not traditional) journals, were available at 19 universities, which were in all cases universities that had an institutional open access policy. Open access publishing is supported by programs such as the German Research Foundation offering financing for open access publishing [33]. However, many institutions still need to develop in this field in terms of greater transparency and access to research, especially with regard to global health aspects. A large percentage of the universities neither had an institutional open access policy nor signed the ‘Berlin Declaration on Open Access to Knowledge in the Sciences and Humanities’, which is seen as a proof of commitment and a mechanism with which to hold universities accountable. Less than a third of the publications were accessible through the PubMed Central corpus, meaning that most German medical faculty global health and poverty-related and neglected diseases research output is likely inaccessible outside of well-financed research institutions.

Equitable licensing policies are university policies that safeguard global access to health products developed through university research by requiring certain clauses to be included in intellectual property licensing agreements that ensure affordability of the product in low- and middle-income countries. This approach has been recommended by the World Health Organization’s Consultative Expert Working Group and has demonstrated successes in Canada and the United States [9, 34]. A study examining equitable licensing practices in the United Kingdom has found a similarly low rate of equitable licensing adoption [5]. Equitable licensing is nearly nonexistent at German medical faculties. Only one institution, the University Medical Center Hamburg-Eppendorf, reported having made use of this legal tool, as part of a research collaboration with the World Health Organization on Ebola research. At nearly all medical faculties surveyed, there are no policies in place to ensure that medicines, diagnostics, or vaccines developed at the faculties are priced affordably in low- and middle-income countries. In order to reflect global health aims such as universal health coverage in their licensing practices, policy changes at German universities are required.

Knowledge on global factors influencing health of individuals and populations will be essential for the future generations of medical doctors [35]. Even though the majority of German medical students endorse the establishment of global health education, the participation in any global health courses among them is only 9% [36]. A recent study on Germany’s global health academic workforce shows German universities are far less equipped in comparison to overseas universities as in the USA or UK [37]. Global health education is offered by the majority of German medical faculties but comprises mostly elective courses (Table 3). This suggests that the students who obtain global health education are primarily those with a pre-existing interest, while the majority of medical students receive no global health teaching.

Only one smaller medical faculty included a compulsory global health course in their curriculum. Considering the high international mobility of German medical students and the swift development of a globalized world, compulsory global health education in the medical curriculum is notably absent and should be integrated. This appeal has been supported for many years by different organizations, such as the German medical students’ association (BVMD), but our findings show that there is still space and need for development. Medical curricula follow state regulations for medical education (Approbationsordnung), a federal directive with the last version in force since 2003 which is required for a fundamental update [38].

A recent study by Kaffes et al. identified 13 medical faculties offering global health education in Germany, a lower number than was identified by our analysis [39]. This discrepancy likely results from the narrower inclusion criteria of Kaffes et al., who only included courses with ‘global health’ or the German translation “globale Gesundheit” in their title, compared to our use of the Consortium of Universities for Global Health definition of global health.

North-South cooperation has been identified by the Organization for Economic Cooperation and Development (OECD) Development Co-operation Directorate and the United Nations Department of Economic and Social Affairs (UNDESA) as an important component of driving the development of research institutions in low- and middle-income countries [40, 41]. We found that approximately half of German medical faculties are involved in research and training partnerships with hospitals and research institutions in low- and middle-income countries. Cooperation could be further strengthened and programs that support cooperation (e.g. the German Academic Exchange Service (DAAD)) could be made more widely known at German universities. Our results regarding student exchange programs in cooperation with low- and middle-income countries show that a majority of German medical faculties supports their students going abroad; in contrast to that, only a low number of programs support students in low- and middle-income countries coming to Germany. Our results suggest a North-South imbalance of opportunities, which should be addressed in the planning of future student exchange opportunities.

### Limitations

In Germany, data on research funding for individual universities are difficult to access and only few universities responded to requests for specific data. This may reflect low priority given to independent research requests, or the lack of established categories such as global health or poverty-related and neglected diseases in research activity reporting, rendering responses time-consuming or impossible, and strongly hints at unavailability of systematic data on global health activities at German universities. Using numerous complementary sources, in most cases, however, we were able to supplement missing and validate gathered data through triangulation.

We assume that our extensive and detailed online searches in public databases and on individual websites of universities, allowed for a comprehensive picture regarding measures of global health access, assuming that universities being engaged in open access publishing and equitable licensing would present this issue on their university website.

While triangulating our online searches on global health education with questionnaires, we were still missing data from less than one third of medical schools. Substantiated by anecdotal evidence gathered through student networks, we assumed that those universities do not provide any global health education.

Most third party funding agencies transparently provide comprehensive funding information. While the German Research Foundation provides detailed information on funded research projects in publicly accessible databases, unfortunately this does not include individual projects grants. Since our repeated requests to the German Research Foundation for grant funding data were frustrated, we therefore approached German Research Foundation grant holders individually for funding information, or aimed to back track grant funding’s through information received from institutions. We acknowledge an unquantifiable unknown in our results on overall research funding’s related to fourty German Research Foundation grants for which no funding information is available.

As a relatively newly emerging field, different global health definitions and the variety of topics considered relevant to global health limit comparability. The search criteria in our study aimed to include a considerable proportion of global health relevant topics, but we acknowledged that we might not have included the entirety of research that could be attributed to global health at medical faculties in Germany.

We are aware that the project scope was limited to research conducted at medical faculties onlyto maintain a manageable project size, and did not include research conducted outside these faculties in departments such as public health, nursing, pharmacy or other allied health fields and in anthropological, geographical, agricultural or socioeconomic development studies that are engaged in global health research. Nevertheless, our results show for the first time the high need to internationally specify the extent of universities’ commitment in both global health education as well as global health and poverty-related and neglected diseases research.

Moreover, global health aspects at medical faculties are not only taught in special courses but can be found as part of other lectures or seminars, e.g. in Public Health, Medical Sociology, Ethics, or even clinical subjects, which makes data collection even more difficult in this field. The lack of a standardized access to the detailed curricula of medical education limited our data acquisition on global health education and made it more dependent on the responsiveness and cooperation of individual persons.

## Conclusions

To our knowledge, this is the first study to analyze global health innovation, access education at German medical faculties, focusing on empiric data related to research and publications and global health related to policies. Data on global health research and education at medical faculties in Germany are neither systematically published nor easily available. At present, significant efforts are required to collect such data and this can be done only with limited specificity and sensitivity, making it difficult to determine the extent of global health activities at German universities in total and in detail. This analysis found that funding for global health and poverty-related and neglected diseases research was low considering the need and was highly concentrated in a small number of institutions, open access publishing policies and equitable licensing policies were mostly absent, and there are little opportunities for global health education. This suggests that global health is not yet established in research and education practice at the majority of medical faculties in Germany.

For Germany to become a strong multi- and bilateral partner in global health and to meet its potential in this area, increased funding, greater commitment and targeted development of human resources in the areas of global health and poverty-related and neglected diseases are needed at medical faculties in Germany and beyond. Universities should openly share information on their work in these areas.

## Supporting information

S1 Appendix Questionnaire in German and English translation. (PDF)

S2 Appendix PubMed advanced search builder search term. (PDF)

S1 Table Requested data. (PDF)

S2 Table Overview of open access publishing at universities in Germany. (PDF)
