# Correction: Global health research and education at medical faculties in Germany

**DOI:** 10.1371/journal.pone.0263556

**Published:** 2022-01-31

**Authors:** Léonie Karduck, Anna Lisa Behnke, Alicia Baier, Dzintars Gotham, Peter Grabitz, Nora Lennartz, Lara Speer, Peter Tinnemann, Walter Bruchhausen

There are errors in the Author Contributions. Anna Lisa Behnke, Alicia Baier, Nora Lennartz and Lara Speer should not have been listed as contributing to “Writing–original draft”.

## References

[1] Karduck L, Behnke AL, Baier A, Gotham D, Grabitz P, Lennartz N, et al. (2020) Global health research and education at medical faculties in Germany. PLoS ONE 15(4): e0231302. 10.1371/journal.pone.0231302 32310987 PMC7170220

