# Supplementary material for: Global health research and education at medical faculties in Germany
Source: PLoS One. 2020 Apr 20;15(4):e0231302. doi: 10.1371/journal.pone.0231302 (PMC7170220; doi:10.1371/journal.pone.0231302)
Supplement: S1 Appendix — (PDF) [file pone.0231302.s001.pdf]

S1 - Fragebogen – Deutsch (English Translation below)

Fragebogen – Innovation & Forschung

1. Bitte geben Sie Ihren Kontakt für eventuelle Rückfragen an. Herzlichen Dank!

Name:
Vorname:
Institution:
Straße:
E-Mail:
Telefon:

2. Wie viel Geld wurde insgesamt an der medizinischen Fakultät durch Drittmittel von 2010 bis 2014 eingeworben?
Bitte Fördersumme in Tausend Euro angeben. (z.B. 35 000 Euro bitte als 35 angeben)

2010:
2011:
2012:
2013:
2014:

3. Welche Forschungsprojekte im Bereich Global Health und/oder armutsassoziierten und vernachlässigten Erkrankungen gab es an der medizinischen Fakultät von 2010 bis 2014?
An dieser Stelle sind ebenfalls Forschungsprojekte von Interesse, welche von der medizinischen Fakultät interdisziplinär mit weiteren Fachdisziplinen durchgeführt werden.

Bitte geben Sie bei Forschungsprojekten, die in Zusammenhang mit Prinzipien des „Open Source Drug Discovery‟ (OSDD) sowie „Produktentwicklungspartnerschaften‟ (PEP) oder „Public-Private Partnerships‟ (PPP) stehen, die jeweiligen Kürzel zum jeweiligen Projekt an.

Bitte machen Sie folgende Angaben pro Forschungsprojekt:

a) Titel/Thema
b) Fördersumme (Bitte Angabe der Fördersumme in Tausend Euro. (z.B. 35 000 Euro bitte als 35 angeben))
c) Geldgeber
d) Förderzeitraum
e) OSDD/PEP/PPP?

4. Wie viele Publikationen wurden von der medizinischen Fakultät von 2010 bis einschließlich 2014 veröffentlicht?
Unter ‛Publikationen‛ verstehen wir alle Journal Article, die einen Peer-Review-Prozess durchlaufen haben und in Zeitschriften mit oder ohne Impact-Faktor publiziert wurden.

Bitte pro Jahr angeben:

2010:
2011:
2012:
2013:
2014:

5. Gibt es an der medizinischen Fakultät Arbeits- oder Organisationsstrukturen, die zu einem erheblichen Teil im Bereich Global Health und/oder armutsassoziierter vernachlässigter Erkrankungen forschen?
z.B. Arbeitsgruppe (≥ 2 Mitarbeiter), Zentrum/ Institut, Lehrstuhl

Bitte machen Sie folgende Angaben zur jeweiligen Struktur:

a) Name
b) Forschungsbereich
c) Tätigkeitszeitraum

6. Gibt es an der medizinischen Fakultät Kooperationen mit Ländern mit niedrigem oder mittlerem Einkommen im Rahmen eines Forschungsaustausches?

Bitte machen Sie folgende Angaben zu Austauschprogrammen in den Jahren 2010 bis 2014.

a) Universität/Klinik
b) Land
c) Beginn (der Zeitpunkt, seitdem die Kooperation besteht)
d) Häufigkeit (die Regelmäßigkeit, in der dieser Austausch stattfindet; z.B. jedes Jahr, alle zwei Jahre)

Fragebogen – Open Access

1. Unterstützt die medizinische Fakultät Ihrer Universität bzw. Ihre Universität als Ganzes Open-Access-Publizieren im Rahmen einer eigenen oder einer gesamtuniversitären Leitlinie?
2. Hat die medizinische Fakultät Ihrer Universität bzw. Ihre Universität als Ganzes eine/n Open-Access-Beauftragte/n ernannt?
Falls „Ja", bitte das Jahr der Ernennung und Kontaktinformationen (Name, E-Mail-Adresse, Telefon) angeben.

3. Richtet die medizinische Fakultät Ihrer Universität bzw. Ihre Universität als Ganzes regelmäßige Informationsveranstaltungen für WissenschaftlerInnen zu Open Access aus?
Falls „Ja", bitte die Frequenz und den Beginn des Angebotes angeben.

4. Stellt die medizinische Fakultät Ihrer Universität bzw. Ihre Universität als Ganzes erschienene Verlagspublikationen und/oder Abschlussarbeiten von Studierenden auf einem Repositorium (frei zugängliche Datenbank) zur Verfügung?
Falls „Ja", bitte das Jahr der Einrichtung des Repositoriums, eine/n AnsprechpartnerIn und die URL angeben.

5. Hat die medizinische Fakultät Ihrer Universität bzw. Ihre Universität als Ganzes eine oder mehrere Open-Access-Zeitschriften im medizinischen Bereich herausgegeben?
Falls „Ja", bitte das Jahr der Gründung, eine/n AnsprechpartnerIn und die URL angeben.

6. Übernimmt die medizinische Fakultät Ihrer Universität bzw. Ihre Universität als Ganzes Publikationsgebühren für Open-Access-Publikationen ihrer Mitglieder?
Falls ja,
a) bis zu welchen Betrag?
b) Welche Bedingungen müssen die AutorInnen, der eingereichte Beitrag und/oder die betreffende Zeitschrift erfüllen, damit die Publikationsgebühren übernommen werden?

7. Wie viel Prozent der Publikationen der medizinischen Fakultät wurden im Zeitraum von 2010-2014 Open Access veröffentlicht?
Unter 'Publikationen' verstehen wir alle Journal Article, die einen Peer-Review-Prozess durchlaufen haben und in Zeitschriften mit oder ohne Impact-Faktor publiziert wurden.

2010 ____ %

2011 ____ %

2012 ____ %

2013 ____ %

2014 ____ %

Fragebogen – Technologietransfer

1. Hat die medizinische Fakultät Ihrer Universität bzw. Ihre Universität als Ganzes in offizieller Form (auf ihrer Webseite oder in internen Dokumenten) Stellung dazu bezogen, ob die Ergebnisse der universitären Forschung durch differenzierte oder nicht-exklusive Lizenzierungsmodelle auch Menschen in Ländern mit niedrigem und mittlerem Einkommen zugänglich gemacht werden sollen?

Bitte entnehmen Sie die Auflistung der Länder mit mittlerem und niedrigem Einkommen der beigefügten Einteilung der Weltbank.

Falls „Ja",

a) seit wann bestehen diese Aussagen?
b) von welchem Gremium innerhalb der Fakultät bzw. Universität wurden sie verabschiedet?
c) welchen Rechtstatus haben sie? (z.B. unverbindliche Erklärung, Teil der Fakultäts- oder Hochschulsatzung)
d) bitte die URL des Dokuments angeben.

2. Hat die medizinische Fakultät Ihrer Universität bzw. Ihre Universität als Ganzes bereits differenzierte oder nicht-exklusive Lizenzierungsmodelle eingesetzt, um Forschungsergebnisse auch Menschen in Ländern mit niedrigem und mittlerem Einkommen zugänglich zu machen?

Bitte entnehmen Sie die Auflistung der Länder mit mittlerem und niedrigem Einkommen der beigefügten Einteilung der Weltbank.

Falls „Ja",

a) in wie viel Prozent aller Lizenzierungen?
b) für welche Innovationen?

3. Für wie viel Prozent der von der medizinischen Fakultät patentierten Innovationen wurden Patente in Ländern mit mittlerem Einkommen angemeldet?

Bitte entnehmen Sie die Auflistung der Länder mit mittlerem Einkommen der beigefügten Einteilung der Weltbank.

2010	%
2011	%
2012	%
2013	%
2014	%

4. Für wie viel Prozent der von der medizinischen Fakultät patentierten Innovationen wurden Patente in Ländern mit niedrigem Einkommen angemeldet?

Bitte entnehmen Sie die Auflistung der Länder mit niedrigem Einkommen der beigefügten Einteilung der Weltbank.

2010	%
2011	%
2012	%

2013	%
2014	%

Fragebogen – Global Health-Ausbildung

1. Bitte geben Sie Ihren Kontakt für eventuelle Rückfragen an. Herzlichen Dank!

Name:
Vorname:
Institution:
Straße:
E-Mail:
Telefon:

2. Wie viele Studierende hat die medizinische Fakultät insgesamt?

WS 2009/10:
SS 2010:
WS 2010/11:
SS 2011:
WS 2011/12:
SS 2012:
WS 2012/13:
SS 2013:
WS 2013/14:
SS 2014:

3. Hat die medizinische Fakultät den Studierenden ein Ausbildungsangebot im Bereich Global Health in den Jahren 2010 bis 2014 angeboten?
Dies kann zum Beispiel ein Angebot in Form von Seminaren, Wahlfächern, Qualifikationsprofilen, Konferenzen oder Ausflügen mit dem Themenschwerpunkt Global Health sein.

Falls "Ja", möchten wir Sie um weitere Informationen zu den einzelnen Veranstaltungen bitten. Bitte beantworten Sie dazu die unten angegebenen Fragen für alle Veranstaltungen vom WS 2009/10 bis zum SS2014.

Sollte Ihnen bereits eine Auflistung der Global Health-Lehre an der medizinischen Fakultät in anderer Form vorliegen (z.B. Curriculum), aus denen die Antworten zu den Fragen hervorgehen, können Sie uns diese selbstverständlich auch per E-Mail oder postalisch zukommen lassen.

a) Titel der Veranstaltung
b) Thema der Veranstaltung (Bitte stellen Sie in ein paar kurzen Sätzen das Thema der Veranstaltung dar)
c) Semesterangabe (In welchem Zeitraum wurde die Veranstaltung angeboten?)
d) Welche Art von Ausbildungsangebot umfasst dies?
   - curriculares Veranstaltungsangebot
      - Wahlpflichtfach / Qualifikationsprofil / sonstige Veranstaltung mit ECTS-Erwerb
      - Schwerpunktcurriculum
      - Pflichtveranstaltung im Curriculum
   - außercurriculares Veranstaltungsangebot
      - freiwilliges Seminar / Veranstaltung ohne ECTS-Erwerb
      - andere Veranstaltung (Ausflüge, Konferenzen, Exkursionen)
      - Vorbereitungskurs für Auslandsfamulaturen
e) Max. Teilnehmerzahl
f) Semesterwochenstunden
   - Pro Studierender:
   - Gesamtes Lehrdeputat:
g) Leistungsnachweis
   - Keiner

- Teilnahmenachweis
- Klausur / mündl. Prüfung / Referat / Hausarbeit
- anderer Leistungsnachweis (z.B. in Form von aktiver Mitarbeit)

h) Verantwortlichkeit
- ehrenamtlich (Studierende, DozentIn)
- Tätigkeit auf eine Hochschulstelle als „Lehrstunden“ anrechenbar
- Sonstiges

i) Weitere Anmerkungen?

4. Gibt es innerhalb des Global Health-Ausbildungsangebotes der medizinischen Fakultät Veranstaltungen, welche in Kooperation mit anderen Fakultäten organisiert werden?

Falls "Ja", bitte folgende Angaben pro Veranstaltung angeben:

a) Titel der Veranstaltung
b) Beteiligte Fakultäten
c) Kooperationszeitraum

5. Gab es an der medizinischen Fakultät im Zeitraum von 2010 bis 2014 die Möglichkeit ein PhD-Programm mit Schwerpunkt im Bereich Global Health und/oder armutsassoziierter und vernachlässigter Erkrankungen zu absolvieren?

6. Gibt es an der Universität einen Studiengang in International Health, Global Health oder Public Health mit Themenschwerpunkten zu globaler Gesundheit?

Falls "Ja", bitte geben Sie folgende Angaben an:

a) Den Namen dieser Studiengänge
b) Das Jahr, seitdem sie angeboten werden an.
c) Haben Studierende der medizinischen Fakultät die Möglichkeit sich für diese Studiengänge einzuschreiben?
d) Stellt die medizinische Fakultät Dozenten, die in die Lehre dieser Studiengänge eingebunden sind? Falls "Ja", seit welchem Semester?

7. Wie viel Prozent der Studienplätze werden für Studierende aus Ländern mit niedrigem und/oder mittlerem Einkommen bereit gestellt?

Bei mehreren Studiengängen, bitte die Angaben mit Kommata trennen und in der Reihenfolge von der obigen Frage nach den Studiengängen auflisten.

WS 2009/10:
SS 2010:
WS 2010/11:
SS 2011:
WS 2011/12:
SS 2012:
WS 2012/13:
SS 2013:
WS 2013/14:
SS 2014:

8. Hat die medizinische Fakultät im Zeitraum von 2010-2014 eigene Studierende für Praktika oder Studienabschnitte an Universitäten und Kliniken in Länder mit niedrigem oder mittlerem Einkommen entsendet?

Dies schließt nur von der Universität selbst angebotene Programme ein (ERASMUS etc.), nicht aber die Programme der Bundesvertretung der Medizinstudierenden in Deutschland (bvmd).

Bitte machen Sie zu jedem Programm im Zeitraum vom WS 2009/10 bis zum SS 2014 folgende Angaben. (Bitte geben Sie bei ‚Beginn‘ den Zeitpunkt an, seitdem die Kooperation besteht, sowie bei ‚Häufigkeit‘ die Regelmäßigkeit in der dieser Austausch stattfindet (z.B. einmalig, jedes Semester))

a) Universität/Klinik
b) Land
c) Beginn
d) Häufigkeit

9. Hat die medizinische Fakultät von 2010-2014 Studierende anderer Universitäten aus Ländern mit niedrigem oder mittlerem Einkommen für Praktika oder Studienabschnitte aufgenommen?
Bitte machen Sie zu jedem Programm im Zeitraum vom WS 2009/10 bis zum SS 2014 folgende Angaben. (Bitte geben Sie bei ‚Beginn‛ den Zeitpunkt an, seitdem die Kooperation besteht, sowie bei ‚Häufigkeit‛ die Regelmäßigkeit in der dieser Austausch stattfindet (z.B. einmalig, jedes Semester))

a) Universität/Klinik
b) Land
c) Beginn
d) Häufigkeit

10. Gibt es an der medizinischen Fakultät Kooperationen mit Ländern mit niedrigem oder mittlerem Einkommen im Rahmen eines klinischen Austausches in der ärztlichen Aus- und Weiterbildung?
Bitte machen Sie folgende Angaben zu Austauschprogrammen in den Jahren 2010 bis 2014.

e) Universität/Klinik
f) Land
g) Beginn (der Zeitpunkt, seitdem die Kooperation besteht)
h) Häufigkeit (die Regelmäßigkeit, in der dieser Austausch stattfindet; z.B. jedes Jahr, alle zwei Jahre)

WS: winter semester
SS: summer semester

Fragebogen Studierende – Global Health-Ausbildung

1. An welcher Universität studierst du?

2. In welchem Studiengang studierst du?

3. In welchem Fachsemester studierst du?

4. Hat die medizinische Fakultät ihren Studierenden ein Ausbildungsangebot im Bereich Global Health in den Jahren 2010 bis 2014 angeboten?
Dies kann zum Beispiel ein Angebot in Form von Seminaren, Wahlfächern, Qualifikationsprofilen, Konferenzen oder Ausflügen mit dem Themenschwerpunkt Global Health sein.

- Ja
- Nein
- Weiß nicht

Du hast angegeben, dass deine Fakultät ein Ausbildungsangebot im Bereich Global Health angeboten hat. Hier möchten wir dich um weitere Informationen zu den einzelnen Veranstaltungen bitten.
Bitte fülle zu jeder Veranstaltung die unten angegebenen Fragen aus.

a) Titel der Veranstaltung
b) Thema der Veranstaltung (Bitte stelle in ein paar kurzen Sätzen das Thema der Veranstaltung dar)
c) Semesterangabe
d) Welche Art von Ausbildungsangebot umfasst dies?
   - curriculares Veranstaltungsangebot
     - Wahlpflichtfach / Qualifikationsprofil / sonstige Veranstaltung mit ECTS-Erwerb
     - Schwerpunktcurriculum
     - Pflichtveranstaltung im Curriculum
   - außercurriculares Veranstaltungsangebot
     - freiwilliges Seminar / Veranstaltung ohne ECTS-Erwerb
     - andere Veranstaltung (Ausflüge, Konferenzen, Exkursionen)
     - Vorbereitungskurs für Auslandsfamulaturen
e) Max. Teilnehmerzahl
f) Zeitlicher Umfang
   - Wie viele Unterrichteinheiten à 45min pro Semester?
g) Leistungsnachweis
   - Keiner
   - Teilnehmernachweis
   - Klausur / mündl. Prüfung / Referat / Hausarbeit
   - Anderer Leistungsnachweis (z.B. in Form von aktiver Mitarbeit)
h) Verantwortlichkeit
   - ehrenamtlich (Studierende, DozentIn)
   - Tätigkeit auf eine Hochschulstelle als „Lehrstunden" anrechenbar
   - Sonstiges

i) Weitere Veranstaltung angeben?

5. Kennst du innerhalb des Global Health Ausbildungsangebotes der medizinischen Fakultät Veranstaltungen, welche in Kooperation mit anderen Fakultäten organisiert werden?
Falls ja, bitte fülle folgende Angaben aus:

d) Titel der Veranstaltung

e) Beteiligte Fakultäten

f) Kooperationszeitraum

6. Gab es an der medizinischen Fakultät im Zeitraum von 2010 bis 2014 die Möglichkeit ein PhD-Programm mit Schwerpunkt im Bereich Global Health und/oder armutsassoziierter und vernachlässigter Erkrankungen zu absolvieren?

7. Wird an deiner Universität ein Studiengang in International Health, Global Health oder Public Health mit Themenschwerpunkten zu globaler Gesundheit angeboten?

- Ja
    - Du hast angegeben, dass an deiner Universität Studiengänge mit Global-Health-Bezug angeboten werden.
        - Bitte gebe hier den Namen dieser Studiengänge an, falls er dir bekannt ist, sowie das Jahr, seitdem sie angeboten werden.
        - Weißt Du, ob die medizinische Fakultät Dozenten stellt, die in die Lehre dieser Studiengänge eingebunden sind?
- Nein
- Weiß nicht

8. Bietet die medizinische Fakultät die Möglichkeit, Praktika oder Studienabschnitte an Universitäten und Kliniken in Ländern mit mittlerem und/oder niedrigem Einkommen zu absolvieren?

- Programm
- Kooperationspartner
- Zeitraum (Angabe Semester)

9. Nimmt die medizinische Fakultät Studierende anderer Universitäten aus Ländern mit mittlerem und/oder niedrigem Einkommen für Praktika oder Studienabschnitte auf?

- Programm
- Kooperationspartner
- Zeitraum (Angabe Semester)

Fragebogen – English

Questionnaire – Innovation: Research funding and publications

1. Please indicate your contact for possible further inquiries. Thank you very much!
Name:
First name:
Institution:
Street:
E-mail:
Phone:

2. How much money was raised at the faculty of medicine from 2010 to 2014? Please indicate the funding amount in thousands of euros. (e.g. 35 000 Euro please state as 35)
2010:
2011:
2012:
2013:
2014:

3. Were there research projects in the field of global health and/or poverty-related and neglected diseases at the faculty of medicine from 2010 to 2014?
At this point, research projects are also of interest, which are carried out by the medical faculty interdisciplinary with other faculties.

For research projects related to the principles of "Open Source Drug Discovery" (OSDD), "Product Development Partnerships" (PEP) or "Public-Private Partnerships" (PPP), please enter the respective abbreviations for the respective project.

Please provide the following information for each research project:
a) Title/Topic
b) Grant amount (Please indicate the grant amount in thousands of euros). (e.g. 35 000 Euro please indicate as 35)
c) Funder
d) Funding period
e) OSDD/PEP/PPP?

4. How many publications were published by the faculty of medicine from 2010 to 2014?
'Publications' include all journal articles that have undergone a peer review process and have been published in journals with or without impact factor.

Please specify per year:
2010:
2011:
2012:
2013:
2014:

5. Are there any working groups or institutions at the faculty of medicine that are largely doing research on global health and/or poverty-related and neglected diseases?
E.g. working group (≥ 2 employees), center/institute, academic chair

Please provide the following information about the respective structure:
a) Name

b) Research area
c) Period of activity

6. Was there any cooperation at the medical faculty with low- or middle-income countries within the framework of a research exchange?
Please provide the following information on exchange programs in the years 2010 to 2014.

a) University/Clinic
b) Country
c) Start (the date since when the cooperation is existing)
d) Frequency (the regularity with which this exchange is taking place; e.g. every year, every two years)

Questionnaire – Open access

1. Does the faculty of medicine or university support open access publishing within its own guideline?
If "Yes", please indicate the year of adoption of the guideline and the URL.

2. Does the faculty of medicine or university have an open access representative?
If "Yes", please indicate the year of the appointment and contact information (Name, E-mail-address, Telephone number).

3. Does the faculty of medicine or university organize regular information events on open access for researchers?
If "Yes", please indicate the frequency and start of the event.

4. Does the faculty of medicine or university make publisher publications and/or students' theses available in a repository (freely accessible database)?
If "Yes", please indicate the year in which the repository was established, a contact person and the URL.

5. Has the faculty of medicine or university published one or more open access journals in the medical field?
If "Yes", please indicate the year of foundation, a contact person and the URL.

6. Does the faculty of medicine or university pay publication fees for open access publications of its members?
If yes,
a) Up to what amount?
b) What conditions must be fulfilled for the publication fees to be paid?

7) What percentage of the medical faculty's publications were published open access between 2010 and 2014?
By 'publications' we mean all journal articles that have undergone a peer review process and have been published in journals with or without an impact factor.
2010:
2011:
2012:
2013:
2014:

Questionnaire – Equitable technology transfer

1. Has the medical faculty or university officially taken a stand (on its website or in internal documents) on whether the results of university research should be made available to people in low- and middle-income countries through differentiated or non-exclusive licensing models?
Please refer to the World Bank's attached classification for a list of low- and middle-income countries.

If "Yes",
a) How long have these statements existed?
b) Which committee within the faculty or university adopted them?
c) What legal status do they have? (e.g. non-binding declaration, part of the faculty or university statutes)
(d) Please specify the URL of the document.

2. Has the medical faculty or university used differentiated or non-exclusive licensing models to make research results available to people in low- and middle-income countries?
Please refer to the World Bank's attached classification for a list of low- and middle-income countries.

If "Yes",
a) In what percentage of all licenses?
b) For which innovations?

3. What percentage of the innovations patented by the medical faculty were registered in middle-income countries?
Please refer to the World Bank's attached classification for a list of middle-income countries.

2010:
2011:
2012:
2013:
2014:

4. What percentage of the innovations patented by the medical faculty were registered in low-income countries?
Please refer to the World Bank's attached classification for a list of low-income countries.

2010:
2011:
2012:
2013:
2014:

Questionnaire – Global health education

1. Please indicate your contact for possible further inquiries. Thank you very much!
Name:
First name:
Institution:
Street:
E-mail:
Phone:

2. How many students does the Faculty of Medicine have in total?
WS 2009/10:
SS 2010:
WS 2010/11:
SS 2011:
WS 2011/12:
SS 2012:
WS 2012/13:
SS 2013:
WS 2013/14:
SS 2014:

3. Did the faculty of medicine offer students global health education training in 2010-2014?
This could be, for example, an offer in the form of seminars, electives, qualification profiles, conferences or excursions focusing on global health.

If yes, we would like to ask you for more information about each event. Please answer the questions below for all events from WS 2009/10 to SS 2014.

If you already have listings of the educational courses regarding global health at the faculty of medicine in another form (such as a curriculum) that provides the answers to the questions, you can also send these to us via email or postal mail.

a) Title of the event
b) Theme of the event (Please describe the topic of the event in a few short sentences)
c) Semester information (In which period was the event offered?)
d) What kind of training offer does this include?

- Curricular event
  - Elective / Qualification profile / Other event with ECTS (European Credit Transfer and Accumulation System) acquisition
  - Focus curriculum
  - Compulsory course in the curriculum
- Extra-curricular event
  - Voluntary seminar / Event without ECTS acquisition
  - Other event (excursions, conferences)

- Preparation course for internships abroad

e) Maximum number of participants
f) Hours per week per semester
- Per student:
- Total teaching load:

g) Proof of performance
- None
- Attendance record
- Written examination / Oral examination / Presentation / Term paper
- Other proof of performance

h) Responsibility
- voluntary (student, lecturer)
- Activity credited as "teaching hours" to a university position
- Other

i) Further information?

4. Are there any events within the global health education of the medical faculty which are organized in cooperation with other faculties?
If "Yes", please provide the following information for each event:
a) Title of the event
b) Participating faculties
c) Cooperation period

5. Was there an opportunity at the faculty of medicine from 2010 to 2014 to complete a PhD program focusing on global health and/or poverty-associated and neglected diseases?

6. Is there a degree program in International Health, Global Health or Public Health at the university with a focus on global health topics?
If "Yes", please provide the following information:
a) Name of degree program
b) Year since it was offered
c) Do students of the faculty of medicine have the opportunity to sign up for this degree program?
d) Does the faculty of medicine provide lecturers who are involved in the teaching of the degree program? If "Yes", since which semester?

7. What percentage of the study places are made available to students from low- and/or middle-income countries?
WS 2009/10:
SS 2010:
WS 2010/11:
SS 2011:
WS 2011/12:
SS 2012:
WS 2012/13:
SS 2013:
WS 2013/14:
SS 2014:

8) Did students of the faculty of medicine have the opportunity to do internships or study periods at universities and clinics in low- or middle-income countries during the period 2010-2014?
This only includes programs offered by the university itself (ERASMUS, etc.).

Please provide the following information for each program in the period from WS 2009/10 to SS 2014.

a) University/Clinic
b) Country
c) Start
d) Frequency

9. Did the faculty of medicine host students from universities situated in low- or middle-income countries for internships or periods of study from 2010-2014?
Please provide the following information for each program in the period from WS 2009/10 to SS 2014.

a) University/Clinic
b) Country
c) Start
d) Frequency

10. Is there any cooperation with low- or middle-income countries at the medical faculty as part of a clinical exchange during medical education and/or residency?
Please provide the following information on exchange programs in the years 2010 to 2014.

a) University/Clinic
b) Country
c) Start
d) Frequency

WS: winter semester
SS: summer semester

Questionnaire students - Global health education

1. At which university do you study?

2. What are you studying?

3. In which semester are you studying?

4. Did the faculty of medicine offer its students global health education between 2010 and 2014?
For example, in the form of seminars, electives, qualification profiles, conferences or excursions focusing on global health.
- Yes
- No
- Don't know

You have indicated that your faculty has offered an education in global health. Here we would like to ask you for further information about the individual events.
Please complete the questions below for each event:
a) Title of the event
b) Theme of the event (Please describe the topic of the event in a few short sentences)
c) Semester information (In which period was the event offered?)
d) What kind of training offer does this include?
- Curricular event
  - Elective / Qualification profile / Other event with ECTS (European Credit Transfer and Accumulation System) acquisition
  - Focus curriculum
  - Compulsory course in the curriculum
- Extra-curricular event
  - Voluntary seminar / Event without ECTS acquisition
  - Other event (excursions, conferences)
  - Preparation course for internships abroad

e) Maximum number of participants
f) Hours per week per semester
- Per student:
- Total teaching load:

g) Proof of performance
- None
- Attendance record
- Written examination / Oral examination / Presentation / Term paper
- Other proof of performance

h) Responsibility
- voluntary (student, lecturer)
- Activity credited as "teaching hours" to a university position
- Other

i) Further information?

5. Do you know of any events within the global health education of the medical faculty which are organized in cooperation with other faculties?
If "Yes", please provide the following information for each event:
a) Title of the event
b) Participating faculties
c) Cooperation period

6. Was there an opportunity at the faculty of medicine from 2010 to 2014 to complete a PhD program focusing on global health and/or poverty-associated and neglected diseases?

7. Is your university offering a degree program in International Health, Global Health or Public Health with a focus on global health topics?

- Yes
    - You have indicated that global health courses are offered at your university. If known,
        - please enter the name of these programs and the year since they were offered)
        - Do you know if the faculty of medicine provides lecturers who are involved in the teaching of these courses?
- No
- Don't know

8. Does the faculty of medicine offer internships or periods of study at universities and clinics in low- and/or middle-income countries?

- Program
- Cooperation partner
- Period (specify semester)

9. Does the faculty of medicine accept students from other universities from low- and/or middle-income countries for internships or periods of study?

- Program
- Cooperation partner
- Period (specify semester)
