# Supplementary material for: Global health research and education at medical faculties in Germany
Source: PLoS One. 2020 Apr 20;15(4):e0231302. doi: 10.1371/journal.pone.0231302 (PMC7170220; doi:10.1371/journal.pone.0231302)
Supplement: S2 Appendix — (PDF) [file pone.0231302.s002.pdf]

S2 Appendix: PubMed Advanced Search Builder search term

"global health"[Title/Abstract] OR "global public health"[Title/Abstract] OR "international health"[Title/Abstract] OR "public health"[Title/Abstract] OR "world health"[Title/Abstract] OR "World Health Organization"[Title/Abstract] OR "health for all"[Title/Abstract] OR "global governance"[Title/Abstract] OR "global health governance"[Title/Abstract] OR "health policy"[Title/Abstract] OR "universal health coverage"[Title/Abstract] OR "millennium development goals"[Title/Abstract] OR "post 2015"[Title/Abstract] OR "Population Health"[Title/Abstract] OR "global mental health"[Title/Abstract] OR "humanitarian aid"[Title/Abstract] OR "development aid"[Title/Abstract] OR "SDH"[Title/Abstract] OR "Social Determinants of Health"[Title/Abstract] OR "Global Health Diplomacy"[Title/Abstract] OR "Human Rights"[Title/Abstract] OR poverty[Title/Abstract] OR "health inequalities"[Title/Abstract] OR globalisation[Title/Abstract] OR "globalization"[Title/Abstract] OR "Climate Health"[Title/Abstract] OR "developing world"[Title/Abstract] OR "developing country"[Title/Abstract] OR "developing countries"[Title/Abstract] OR "LMIC"[Title/Abstract] OR "lower middle income country"[Title/Abstract] OR "lower-income"[Title/Abstract] OR "middle-income"[Title/Abstract] OR "Transnational"[Title/Abstract] OR "West Africa"[Title/Abstract] OR "east Africa"[Title/Abstract] OR "tropical africa"[Title/Abstract] OR "sub-saharan africa"[Title/Abstract] OR "southeast asia"[Title/Abstract] OR "south-east asia"[Title/Abstract] OR "latin america"[Title/Abstract] OR "south america"[Title/Abstract] OR (trade[Title/Abstract] AND "health"[Title/Abstract]) OR ("climate change"[Title/Abstract] AND "health"[Title/Abstract]) OR "Afghanistan"[Title/Abstract] OR "Benin"[Title/Abstract] OR "Burkina Faso"[Title/Abstract] OR "Burundi"[Title/Abstract] OR "Cambodia"[Title/Abstract] OR "Central African Republic"[Title/Abstract] OR "Chad"[Title/Abstract] OR "Comoros"[Title/Abstract] OR "Congo"[Title/Abstract] OR "Eritrea"[Title/Abstract] OR "Ethiopia"[Title/Abstract] OR "Gambia"[Title/Abstract] OR "Guinea"[Title/Abstract] OR "Guinea-Bissau"[Title/Abstract] OR "Haiti"[Title/Abstract] OR "Korea"[Title/Abstract] OR "Republic Korea"[Title/Abstract] OR "Liberia"[Title/Abstract] OR "Madagascar"[Title/Abstract] OR "Malawi"[Title/Abstract] OR "Mali"[Title/Abstract] OR "Mozambique"[Title/Abstract] OR "Nepal"[Title/Abstract] OR "Niger"[Title/Abstract] OR "Rwanda"[Title/Abstract] OR "Sierra Leone"[Title/Abstract] OR "Somalia"[Title/Abstract] OR "South Sudan"[Title/Abstract] OR "Tanzania"[Title/Abstract] OR "Togo"[Title/Abstract] OR "Uganda"[Title/Abstract] OR "Zimbabwe"[Title/Abstract] OR "Armenia"[Title/Abstract] OR "Bangladesh"[Title/Abstract] OR "Bhutan"[Title/Abstract] OR "Bolivia"[Title/Abstract] OR "Cabo Verde"[Title/Abstract] OR "Cameroon"[Title/Abstract] OR "Congo"[Title/Abstract] OR "Cote d'Ivoire"[Title/Abstract] OR "Djibouti"[Title/Abstract] OR "Egypt"[Title/Abstract] OR "El Salvador"[Title/Abstract] OR "Georgia"[Title/Abstract] OR "Ghana"[Title/Abstract] OR "Guatemala"[Title/Abstract] OR "Guyana"[Title/Abstract] OR "Honduras"[Title/Abstract] OR "India"[Title/Abstract] OR "Indonesia"[Title/Abstract] OR "Kenya"[Title/Abstract] OR "Kiribati"[Title/Abstract] OR "Kosovo"[Title/Abstract] OR "Kyrgyz Republic"[Title/Abstract] OR "Lao"[Title/Abstract] OR "Lesotho"[Title/Abstract] OR "Mauritania"[Title/Abstract] OR "Micronesia"[Title/Abstract] OR "Moldova"[Title/Abstract] OR "Morocco"[Title/Abstract] OR "Myanmar"[Title/Abstract] OR "Nicaragua"[Title/Abstract] OR "Nigeria"[Title/Abstract] OR "Pakistan"[Title/Abstract] OR "Papua New Guinea"[Title/Abstract] OR "Philippines"[Title/Abstract] OR "Samoa"[Title/Abstract] OR "Sao Tome"[Title/Abstract] OR "Sao Tome"[Title/Abstract] OR "Senegal"[Title/Abstract] OR "Solomon Islands"[Title/Abstract] OR "Sri Lanka"[Title/Abstract] OR "Sudan"[Title/Abstract] OR "Swaziland"[Title/Abstract] OR "Syrian"[Title/Abstract] OR "Tajikistan"[Title/Abstract] OR "Timor-Leste"[Title/Abstract] OR "Timor Leste"[Title/Abstract] OR "Ukraine"[Title/Abstract] OR "Uzbekistan"[Title/Abstract] OR "Vanuatu"[Title/Abstract] OR "Vietnam"[Title/Abstract] OR "West Bank"[Title/Abstract] OR "Gaza"[Title/Abstract] OR "Yemen"[Title/Abstract] OR "Zambia"[Title/Abstract] OR "Albania"[Title/Abstract] OR "Algeria"[Title/Abstract] OR "American Samoa"[Title/Abstract] OR "Angola"[Title/Abstract] OR "Azerbaijan"[Title/Abstract] OR "Belarus"[Title/Abstract] OR "Belize"[Title/Abstract] OR "Bosnia"[Title/Abstract] OR "Herzegovina"[Title/Abstract] OR "Botswana"[Title/Abstract] OR "Brazil"[Title/Abstract] OR "Bulgaria"[Title/Abstract] OR "China"[Title/Abstract] OR "Colombia"[Title/Abstract] OR "Costa Rica"[Title/Abstract] OR "Cuba"[Title/Abstract] OR "Dominica"[Title/Abstract] OR "Dominican Republic"[Title/Abstract] OR "Ecuador"[Title/Abstract] OR "Fiji"[Title/Abstract] OR "Gabon"[Title/Abstract] OR "Grenada"[Title/Abstract] OR "Iran"[Title/Abstract] OR "Iraq"[Title/Abstract] OR "Jamaica"[Title/Abstract] OR "Jordan"[Title/Abstract] OR "Kazakhstan"[Title/Abstract] OR "Lebanon"[Title/Abstract] OR

"Libya"[Title/Abstract] OR "Macedonia"[Title/Abstract] OR "Malaysia"[Title/Abstract] OR "Maldives"[Title/Abstract] OR "Marshall Islands"[Title/Abstract] OR "Mauritius"[Title/Abstract] OR "Mexico"[Title/Abstract] OR "Mongolia"[Title/Abstract] OR "Montenegro"[Title/Abstract] OR "Namibia"[Title/Abstract] OR "Palau"[Title/Abstract] OR "Panama"[Title/Abstract] OR "Paraguay"[Title/Abstract] OR "Peru"[Title/Abstract] OR "Romania"[Title/Abstract] OR "Serbia"[Title/Abstract] OR "South Africa"[Title/Abstract] OR "St. Lucia"[Title/Abstract] OR "Suriname"[Title/Abstract] OR "Thailand"[Title/Abstract] OR "Tonga"[Title/Abstract] OR "Tunisia"[Title/Abstract] OR "Turkey"[Title/Abstract] OR "Turkmenistan"[Title/Abstract] OR "Tuvalu"[Title/Abstract] OR "Afghanistan"[Title/Abstract] OR "Ruanda"[Title/Abstract] OR "Kongo"[Title/Abstract] OR "Sierra Leone"[Title/Abstract] OR "Benin"[Title/Abstract] OR "Korea"[Title/Abstract] OR "Burkina Faso"[Title/Abstract] OR "Liberia"[Title/Abstract] OR "Somalia"[Title/Abstract] OR "Burundi"[Title/Abstract] OR "Madagaskar"[Title/Abstract] OR "Eritrea"[Title/Abstract] OR "Malawi"[Title/Abstract] OR "Tansania"[Title/Abstract] OR "Gambia"[Title/Abstract] OR "Mali"[Title/Abstract] OR "Togo"[Title/Abstract] OR "Guinea"[Title/Abstract] OR "Guinea-Bissau"[Title/Abstract] OR "Nepal"[Title/Abstract] OR "Uganda"[Title/Abstract] OR "Haiti"[Title/Abstract] OR "Niger"[Title/Abstract] OR "Ruanda"[Title/Abstract] OR "Kamerun"[Title/Abstract] OR "Kenia"[Title/Abstract] OR "Bangladesh"[Title/Abstract] OR "Sambia"[Title/Abstract] OR "Bhutan"[Title/Abstract] OR "Kiribati"[Title/Abstract] OR "Samoa"[Title/Abstract] OR "Kongo"[Title/Abstract] OR "Cabo Verde"[Title/Abstract] OR "Kosovo"[Title/Abstract] OR "Senegal"[Title/Abstract] OR "Cote d'Ivoire"[Title/Abstract] OR "Laos"[Title/Abstract] OR "Sri Lanka"[Title/Abstract] OR "Lesotho"[Title/Abstract] OR "Sudan"[Title/Abstract] OR "El Salvador"[Title/Abstract] OR "Marokko"[Title/Abstract] OR "Ghana"[Title/Abstract] OR "Guatemala"[Title/Abstract] OR "Myanmar"[Title/Abstract] OR "Timor-Leste"[Title/Abstract] OR "Guyana"[Title/Abstract] OR "Nicaragua"[Title/Abstract] OR "Ukraine"[Title/Abstract] OR "Honduras"[Title/Abstract] OR "Nigeria"[Title/Abstract] OR "Usbekistan"[Title/Abstract] OR "Indien"[Title/Abstract] OR "Pakistan"[Title/Abstract] OR "Vanuatu"[Title/Abstract] OR "Vietnam"[Title/Abstract] OR "Jemen"[Title/Abstract] OR "West Bank"[Title/Abstract] OR "Gaza"[Title/Abstract] OR "Gabun"[Title/Abstract] OR "Namibia"[Title/Abstract] OR "Algerien"[Title/Abstract] OR "Grenada"[Title/Abstract] OR "Palau"[Title/Abstract] OR "Irak"[Title/Abstract] OR "Panama"[Title/Abstract] OR "Angola"[Title/Abstract] OR "Iran"[Title/Abstract] OR "Paraguay"[Title/Abstract] OR "Peru"[Title/Abstract] OR "Belarus"[Title/Abstract] OR "Rumanien"[Title/Abstract] OR "Belize"[Title/Abstract] OR "Kasachstan"[Title/Abstract] OR "Herzegowina"[Title/Abstract] OR "St. Lucia"[Title/Abstract] OR "Kuba"[Title/Abstract] OR "St. Vincent"[Title/Abstract] OR "Brasilien"[Title/Abstract] OR "Libanon"[Title/Abstract] OR "Suriname"[Title/Abstract] OR "China"[Title/Abstract] OR "Malaysia"[Title/Abstract] OR Thailand[Title/Abstract] OR "Costa Rica"[Title/Abstract] OR Tonga[Title/Abstract] OR Dominica[Title/Abstract] OR Mauritius[Title/Abstract] OR Turkei[Title/Abstract] OR Ecuador[Title/Abstract] OR Turkmenistan[Title/Abstract] OR Tuvalu[Title/Abstract] OR Montenegro[Title/Abstract] OR "Neglected Tropical Disease"[Title/Abstract] OR hiv[Title/Abstract] OR "human immunodeficiency virus"[Title/Abstract] OR malaria[Title/Abstract] OR plasmodium[Title/Abstract] OR tuberculosis[Title/Abstract] OR Tuberkulose[Title/Abstract] OR TB[Title/Abstract] OR "mycobacterium tuberculosis"[Title/Abstract] OR "M tuberculosis"[Title/Abstract] OR "mycobacterium bovis"[Title/Abstract] OR "M bovis"[Title/Abstract] OR ascaridiasis[Title/Abstract] OR ascariasis[Title/Abstract] OR "roundworm infection"[Title/Abstract] OR Ancylostoma[Title/Abstract] OR "Necator americanus"[Title/Abstract] OR "Hookworm disease"[Title/Abstract] OR uncinariasis[Title/Abstract] OR Ancylostomiasis[Title/Abstract] OR Necatoriasis[Title/Abstract] OR Trichuriasis[Title/Abstract] OR trichocephaliasis[Title/Abstract] OR whipworm[Title/Abstract] OR Strongyloidiasis[Title/Abstract] OR trichostrongyliasis[Title/Abstract] OR "intestinal roundworm"[Title/Abstract] OR Filariose[Title/Abstract] OR "Wuchereria bancrofti"[Title/Abstract] OR Elephantiasis[Title/Abstract] OR filariasis[Title/Abstract] OR "Bancroftian elephantiasis"[Title/Abstract] OR "Bancroftian filariasis"[Title/Abstract] OR Onchocerciasis[Title/Abstract] OR "onchocerca volvulus infection"[Title/Abstract] OR onchocercosis[Title/Abstract] OR "river blindness"[Title/Abstract] OR Schistosomiasis[Title/Abstract] OR Bilharziose[Title/Abstract] OR bilharziasis[Title/Abstract] OR Schistosoma[Title/Abstract] OR Cysticercosis[Title/Abstract] OR "Taenia solium"[Title/Abstract] OR "T solium"[Title/Abstract] OR taeniasis[Title/Abstract] OR chagas[Title/Abstract] OR "American trypanosomiasis"[Title/Abstract] OR "trypanosoma cruzi"[Title/Abstract] OR "T cruzi"[Title/Abstract] OR "Trypanosoma brucei"[Title/Abstract] OR "T brucei"[Title/Abstract] OR "African trypanosomiasis"[Title/Abstract] OR "african sleeping

sickness"[Title/Abstract] OR "Gambiense trypanosomiasis"[Title/Abstract] OR "Rhodesiense trypanosomiasis"[Title/Abstract] OR leishmaniose[Title/Abstract] OR leishmaniasis[Title/Abstract] OR "kala-azar"[Title/Abstract] OR espundia[Title/Abstract] OR Rotavirus[Title/Abstract] OR "Rotaviral enteritis"[Title/Abstract] OR ETEC[Title/Abstract] OR "enterotoxigenic escherichia coli"[Title/Abstract] OR "enterotoxigenic e coli"[Title/Abstract] OR cholera[Title/Abstract] OR "vibrio cholerae"[Title/Abstract] OR "V. cholerae"[Title/Abstract] OR "V cholerae"[Title/Abstract] OR shigella[Title/Abstract] OR shigellosis[Title/Abstract] OR cryptosporidiosis[Title/Abstract] OR EAggEC[Title/Abstract] OR "enteroaggregative escherichia coli"[Title/Abstract] OR giardiasis[Title/Abstract] OR lambliasis[Title/Abstract] OR "giardia infection"[Title/Abstract] OR dengue[Title/Abstract] OR "meningococcal meningitis"[Title/Abstract] OR Typhus[Title/Abstract] OR Paratyphus[Title/Abstract] OR Salmonella[Title/Abstract] OR Salmonellen[Title/Abstract] OR "typhoid fever"[Title/Abstract] OR "paratyphoid fever"[Title/Abstract] OR "hepatitis C"[Title/Abstract] OR HCV[Title/Abstract] OR lepra[Title/Abstract] OR "mycobacterium leprae"[Title/Abstract] OR "m. leprae"[Title/Abstract] OR leprosy[Title/Abstract] OR "hansen's disease"[Title/Abstract] OR buruli[Title/Abstract] OR "mycobacterium ulcerans"[Title/Abstract] OR "M ulcerans"[Title/Abstract] OR trachoma[Title/Abstract] OR "chlamydia trachomatis"[Title/Abstract] OR "c trachomatis"[Title/Abstract] OR "Cryptococcal meningitis"[Title/Abstract] OR "rheumatic fever"[Title/Abstract] OR Leptospirose[Title/Abstract] OR Leptospirosis[Title/Abstract] OR ("streptococcus pneumoniae"[Title/Abstract] AND pneumonia[Title/Abstract]) OR ("streptcoccus pneumoniae"[Title/Abstract] AND pneumonie[Title/Abstract]) OR ("S. pneumoniae"[Title/Abstract] AND pneumonia[Title/Abstract]) OR ("S. pneumoniae"[Title/Abstract] AND pneumonie[Title/Abstract]) OR Meningokokkenmeningitis[Title/Abstract] OR (meningitis[Title/Abstract] AND "neisseria meningitidis"[Title/Abstract]) OR (meningitis[Title/Abstract] AND "N. meningitidis"[Title/Abstract])
