# Supplementary material for: Global health research and education at medical faculties in Germany
Source: PLoS One. 2020 Apr 20;15(4):e0231302. doi: 10.1371/journal.pone.0231302 (PMC7170220; doi:10.1371/journal.pone.0231302)
Supplement: S1 Table — (PDF) [file pone.0231302.s003.pdf]

Medical faculty of	University response	Student represetatives response	Global health education? (Regularity? More than one type of GH education? More than two events? Included in compulsory medical curriculum?)			Interdisciplinary GH courses with other faculties?			Exchange-program with LMICs?			Research and/or training partnerships with LMICs?	
		Number of completed questionnaires	University response	Student represetatives response	Online data search	University response	Student represetatives response	Online data search	University response	Student represetatives response	Online data search	University response	Online data search
University of Aachen	response to questionnaire and contact person indicated	2	yes	yes	no online data search	no response	no or no response	no online data search	no response	outgoing-program	outgoing-program	no response	no variable data found
Charité - Universitätsmedizin Berlin	no response	34	no response	yes	no online data search	no response	no or no response	no online data search	no response	no response	outgoing--program	no response	yes
Ruhr University Bochum	no response	1	no response	not known by student	implemented, but no GHE was found	no response	no or no response	no data found	no response	no response	inclusion criteria not	no response	no variable data found
University of Bonn	response to questionnaire or via e-mail and contact person indicated	1	yes	not known by student	no online data search	yes	no or no response	no online data search	incoming- and outgoing-program	no response	no online data search	yes	no variable data found
Dresden University of Technology	no response	2	no response	no	GHE was found	no response	no or no response	no data found	no response	no response	outgoing-program	no response	no variable data found
Heinrich-Heine University Düsseldorf	response to questionnaire or via e-mail	1	response, but data unable to verify	no	GHE was found	no response	no or no response	no data found	no response	no response	outgoing-program	no response	yes
Friedrich-Alexander University Erlangen-Nürnberg	no response	0	no response	no response	GHE was found, but data outside the temporal inclusion	no response	no or no response	no data found	no response	no response	outgoing-program	no response	no variable data found
University of Duisburg-Essen	no response	0	no response	no response	implemented, but no GHE was found	no response	no or no response	no data found	no response	no response	outgoing-program	no response	no variable data found
Goethe University Frankfurt	no response	2	no response	yes, but data unable to verify	GHE was found, but unable to verify	no response	no or no response	no data found	no response	no response	outgoing-program	no response	yes
University of Freiburg	response to questionnaire or via e-mail and contact person indicated	4	yes	yes	no online data search	yes, but data outside the temporal inclusion	no or no response	no online data search	outgoing-program	no response	no online data search	yes	no variable data found
University of Giessen	no response	8	no response	yes	GHE was found	no response	yes	no online data search	no response	inclusion criteria not	inclusion criteria not	no response	no variable data found
University of Göttingen	no response	0	no response	no response	GHE was found, but data outside the temporal inclusion	no response	no or no response	no data found	no response	no response	inclusion criteria not met	no response	yes
University of Greifswald	response to questionnaire or via e-mail and contact person indicated	14	yes	yes, but data unable to verify	no online data search	no response	yes	no online data search	no response	no response	no online data search	yes	no variable data found
Martin Luther University of Halle-Wittenberg	no response	14	no response	yes	no online data search	no response	no or no response	no online data search	no response	no response	no data found	no response	yes
University of Hamburg	response to questionnaire or via e-mail and contact person indicated	8	yes	not known by students	GHE was found	data unable to verify	no or no response	no online data search	incoming- and outgoing-program	no response	no online data search	no variable data available	yes
Hannover Medical School	Reply to questionnaire rejected	21	no response	no or not known by students	no online data search	no response	no or no response	no online data search	no response	no response	no online data search	no response	no variable data found
Heidelberg University / Medical Faculty Heidelberg	response to questionnaire or via e-mail and contact person indicated	2	yes	no or not known by students	GHE was found, but unable to verify	no response	no or no response	data unable to verify	no response	no response	incoming- and outgoing-program	no response	yes
University of Saarland	Reply to questionnaire rejected	3	no response	yes, but unable to verify	implemented, but no GHE was found	no response	no or no response	no data found	no response	no response	no data found	no response	no variable data found
Friedrich Schiller University Jena	no response	0	no response	no response	GHE was found	no response	no or no response	no data found	no response	no response	incoming- and outgoing-	no response	yes
Christian Albrechts University Kiel	no response	20	no response	yes	no online data search	no response	no or no response	no online data search	no response	unable to verify	no online data search	no response	no variable data found

University of Cologne	no response	2	no response	no or not known by students	implemented, but no GHE was found	no response	no or no response	no data found	no response	no response	outgoing-program	no response	yes
University of Leipzig	response to questionnaire or via e-mail and contact person indicated	0	yes	no response	no online data search	no response	no or no response	no online data search	no response	no response	outgoing-program	no response	no variable data found
University of Lübeck	no response	0	no response	no response	implemented, but no GHE was found	no response	no or no response	no data found	no response	no response	outgoing-program	no response	yes
Otto-von-Guericke University Magdeburg	response to questionnaire or via e-mail and contact person indicated	0	yes	no response	no online data search	no response	no or no response	no online data search	outgoing-program	no response	no online data search	yes, but outside the teporal inclusion criteria	no variable data found
Heidelberg University / Medical Faculty Mannheim	no response	0	no response	no response	GHE was found, but unable to verify	no response	no or no response	no data found	no response	no response	inclusion criteria not	no response	no variable data found
Johannes Gutenberg University of Mainz	no response	22	no response	no or not known by students	no online data search	no response	no or no response	no online data search	no response	incoming- and outgoing	no online data search	no response	no variable data found
Philipps University of Marburg	response to questionnaire or via e-mail and contact person indicated	1	yes	no	GHE was found	no response	no or no response	no online data search	no response	no response	no online data search	no response	no variable data found
Ludwig Maximilians University of Munich	contact person indicated	1	yes	no	GHE was found	response but unable to	no or no response	no data found	incoming- and outgoing-	no response	incoming- and outgoing-	yes	yes
Technical University of Munich	Reply to questionnaire rejected	44	no response	yes	no online data search	no response	no or no response	no online data search	no response	no response	no online data search	no response	yes
University of Münster	no response	3	no response	yes	GHE was found, but data outside the temporal inclusion	no response	no or no response	data unable to verify	no response	data unable to verify	outgoing-program	no response	yes
Carl von Ossietzky University of Oldenburg	response to questionnaire or via e-mail and contact person indicated	2	yes	yes	no online data search	no	no or no response	no online data search	inclusion criteria not met	outgoing-program	no online data search	not yet implemented	no variable data found
University of Regensburg	no response	9	no response	yes	implemented, but no GHE was found	no response	no or no response	no data found	no response	no response	inclusion criteria not	no response	no variable data found
University of Rostock	no response	0	no response	no response	implemented, but data unable to	no response	no or no response	no data found	no response	no response	outgoing-program	no response	yes
University of Tübingen	Reply to questionnaire rejected	0	no response	no response	implemented, but data unable to	no response	no or no response	no data found	no response	no response	outgoing-program	no response	yes
University of Ulm	response to questionnaire or via e-mail and contact person indicated	1	yes	yes, but unable to verify	no online data search	no response	no or no response	no online data search	no response	no response	no online data search	yes	no variable data found
University of Würzburg	contact person indicated	0	no response	no response	GHE was found	no response	data unable to verify	yes	no response	no response	outgoing-program	no response	no variable data found
