# Supplementary material for: Global health research and education at medical faculties in Germany
Source: PLoS One. 2020 Apr 20;15(4):e0231302. doi: 10.1371/journal.pone.0231302 (PMC7170220; doi:10.1371/journal.pone.0231302)
Supplement: S2 Table — (PDF) [file pone.0231302.s004.pdf]

S4 Table: Overview of Open Access Publishing at universities in Germany

University	OA policy	Berlin declaration	OA representative	Website	OA fund	Repository
University of Leipzig	yes	yes	OA representative mentioned	detailed OA information on webpage	yes	yes[a]
Ruhr University Bochum	yes	yes	OA representative mentioned	detailed OA information on webpage	yes	yes[b]
Johannes Gutenberg University of Mainz	yes	yes	OA representative mentioned	detailed OA information on webpage	yes	no
Heinrich-Heine University Düsseldorf	yes	yes	OA representative mentioned	detailed OA information on webpage	yes	yes[a]
University of Ulm	yes	yes	OA representative mentioned	detailed OA information on webpage	yes	no
University of Münster	yes	yes	OA representative mentioned	detailed OA information on webpage	yes	yes[b]
Friedrich-Alexander University Erlangen-Nürnberg	yes	yes	OA representative mentioned	detailed OA information on webpage	yes	yes[b]
Julius Maximilians University of Würzburg	yes	yes	OA representative mentioned	detailed OA information on webpage	yes	yes[b]
Christian Albrechts University Kiel	yes	yes	OA representative mentioned	detailed OA information on webpage	no	yes[b]
Heidelberg University (Medical Faculty Heidelberg)	yes	no	OA representative mentioned	detailed OA information on webpage	yes	yes[b]
Technical university of Munich	yes	no	OA representative mentioned	detailed OA information on webpage	yes	yes[b]
Heidelberg University (Medical Faculty Mannheim)	yes	no	OA representative mentioned	detailed OA information on webpage	yes	yes[b]
Eberhard-Karls University Tübingen	yes	no	OA representative mentioned	detailed OA information on webpage	yes	yes[b]
University of Göttingen	yes	no	contact person mentioned	detailed OA information on webpage	yes	yes[b]
University of Giessen	yes	no	OA representative mentioned	detailed OA information on webpage	yes	yes[b]
Dresden University of Technology	yes	no	OA representative mentioned	detailed OA information on webpage	yes	yes[a]
University of Duisburg-Essen	yes	no	contact person mentioned	detailed OA information on webpage	yes	yes[b]
University of Regensburg	yes	no	OA representative mentioned	detailed OA information on webpage	yes	yes[b]
Albert Ludwig University of Freiburg	yes	no	OA representative mentioned	detailed OA information on webpage	yes	yes[b]
Hannover Medical School	yes	no	contact person mentioned	detailed OA information on webpage	yes	no
University of Saarland	no	yes	no representative	link to external OA webpages + some OA information	no	yes[b]
University of Hamburg	no	no	OA representative mentioned	no	no	yes[b]

Ludwig-Maximilians University of Munich	no	no	e-mail address or telephone number	detailed OA information on webpage	no	yes[b]
University of Aachen	no	no	e-mail address or telephone number	detailed OA information on webpage	no	yes[b]
University of Cologne	no	no	no representative	link to external OA webpages + some OA information	no	yes[b]
Goethe University Frankfurt	no	no	no representative	link to external OA webpages + some OA information	no	yes[b]
University of Rostock	no	no	no representative	link to external OA webpages + some OA information	no	yes[a]
Charité - Universitätsmedizin Berlin	no	no	no representative	link to external OA webpages	no	no
University of Bonn	no	no	e-mail address or telephone number	detailed OA information on webpage	no	no
Friedrich Schiller University Jena	no	no	no representative	link to external OA webpages + some OA information	no	yes[a]
Carl von Ossietzky University of Oldenburg	no	no	OA representative mentioned	detailed OA information on webpage	no	no
Philipps University of Marburg	no	no	no representative	detailed OA information on webpage	no	no
University of Greifswald	no	no	no representative	link to external OA webpages + some OA information	no	no
Otto-von-Guericke University Magdeburg	no	no	no representative	no	no	no
Martin Luther University of Halle-Wittenberg	no	no	no representative	no	no	no
University of Lübeck	no	no	no representative	no	no	no

a Interface in German but English articles included

b Interface in English and English articles included
